# Effects of Source- versus Household Contamination of Tubewell Water on Child Diarrhea in Rural Bangladesh: A Randomized Controlled Trial

**DOI:** 10.1371/journal.pone.0121907

**Published:** 2015-03-27

**Authors:** Ayse Ercumen, Abu Mohd. Naser, Leanne Unicomb, Benjamin F. Arnold, John M. Colford Jr., Stephen P. Luby

**Affiliations:** 1 Division of Epidemiology, School of Public Health, University of California, Berkeley, California, United States of America; 2 Centre for Communicable Diseases, International Centre for Diarrhoeal Disease Research, Dhaka, Bangladesh; 3 School of Medicine, Stanford University, Stanford, California, United States of America; 4 Centers for Disease Control and Prevention, Atlanta, Georgia, United States of America; Johns Hopkins Bloomberg School of Public Health, UNITED STATES

## Abstract

**Background:**

Shallow tubewells are the primary drinking water source for most rural Bangladeshis. Fecal contamination has been detected in tubewells, at low concentrations at the source and at higher levels at the point of use. We conducted a randomized controlled trial to assess whether improving the microbiological quality of tubewell drinking water by household water treatment and safe storage would reduce diarrhea in children <2 years in rural Bangladesh.

**Methods:**

We randomly assigned 1800 households with a child aged 6-18 months (index child) into one of three arms: chlorine plus safe storage, safe storage and control. We followed households with monthly visits for one year to promote the interventions, track their uptake, test participants’ source and stored water for fecal contamination, and record caregiver-reported child diarrhea prevalence (primary outcome). To assess reporting bias, we also collected data on health outcomes that are not expected to be impacted by our interventions.

**Findings:**

Both interventions had high uptake. Safe storage, alone or combined with chlorination, reduced heavy contamination of stored water. Compared to controls, diarrhea in index children was reduced by 36% in the chlorine plus safe storage arm (prevalence ratio, PR = 0.64, 0.55-0.73) and 31% in the safe storage arm (PR = 0.69, 0.60-0.80), with no difference between the two intervention arms. One limitation of the study was the non-blinded design with self-reported outcomes. However, the prevalence of health outcomes not expected to be impacted by water interventions did not differ between study arms, suggesting minimal reporting bias.

**Conclusions:**

Safe storage significantly improved drinking water quality at the point of use and reduced child diarrhea in rural Bangladesh. There was no added benefit from combining safe storage with chlorination. Efforts should be undertaken to implement and evaluate long-term efforts for safe water storage in Bangladesh.

**Trial Registration:**

ClinicalTrials.gov NCT01350063

## Introduction

The majority of rural Bangladeshis obtain drinking water from groundwater aquifers using shallow tubewells [1]. Groundwater is typically considered microbiologically safe due to natural pathogen removal and inactivation by percolation through soil [2]. However, studies conducted during the period of widespread tubewell installation in Bangladesh in the 1970s failed to detect reductions in rates of cholera and other diarrheal diseases in tubewell users vs. users of fecally contaminated surface water sources [3–6].

One possible explanation for the failure of tubewells to prevent diarrhea could be that tubewell water remains sufficiently contaminated with pathogens to pose a health risk. Fecal contamination has been detected in groundwater sources from various settings in developed as well as developing countries [7–9]. Recent studies in Bangladesh have demonstrated that up to 65% of tubewells can contain indicators of fecal contamination such as fecal/thermotolerant coliforms and *Escherichia coli (E*. *coli*); the level of contamination, however, is typically low [10–17]. Fecal pathogens including rotavirus, adenovirus, *Shigella*, *Vibrio cholerae* and enterotoxigenic *E*. *coli* have also been detected in tubewell water [10,14]. Tubewells in rural Bangladesh are often located in close proximity to latrines and ponds. Possible mechanisms for tubewell contamination with fecal pathogens include infiltration into the groundwater aquifers from nearby latrines, septic tanks and ponds [18,19], short-circuiting of contaminated surface water into the wells through unsealed tubewell components [14], or harboring of bacteria in contaminated handpumps [20].

An alternative explanation for the lack of a reduction in diarrhea after tubewell installation could be that, although tubewell water may be relatively safe at the source, it becomes contaminated during collection, handling and storage in households. On average, 55% of rural Bangladeshis store water for drinking and cooking purposes, depending on season and the proximity of the tubewell to the kitchen [12]. The most commonly used storage container is the *kolshi*; a lidless aluminum vessel with a narrow mouth but a wide brim, which leaves water vulnerable to contamination by contact with hands [21]. Point-of-use contamination of drinking water during storage in households has been well-documented in various settings, and is a major factor leading to deterioration of drinking water quality in environments where the source water quality is relatively good, such as in the case of tubewell water in Bangladesh [22,23]. In rural Bangladesh, point-of-use contamination of stored water is common [12], and the presence of fecal indicators in stored water (as opposed to in source water) has been associated with diarrhea risk among children [24].

An estimated 16,600 to 27,700 children die each year in Bangladesh from diarrheal disease [25]. As a large majority of Bangladeshis rely on tubewells for drinking water, the issue of whether and to what extent contamination of tubewell water at the source or at the point of use contributes to this disease burden is a critically important question. In addition to microbial contamination, groundwater in many regions of Bangladesh is also contaminated with high levels of naturally occurring arsenic [26]. There is high spatial variation in the distribution of groundwater arsenic, and therefore a common mitigation method for reducing exposure is for a family with a contaminated tubewell to switch to a nearby, arsenic-free tubewell [27]. However, recent research indicates that arsenic concentration in shallow tubewells is inversely related to microbiological contamination [11,15]. This makes the provision of safe drinking water in Bangladesh particularly difficult. As families switch to low-arsenic wells with potentially higher levels of fecal contamination and inadvertently put themselves at risk of increased pathogen exposure [28], assessing whether taking additional steps such as treating and safely storing tubewell drinking water can effectively reduce diarrhea in this setting is of particular importance in ensuring access to safe drinking water.

We conducted a randomized controlled trial to evaluate the individual and combined impact of safely storing and chlorinating tubewell water on household water quality and diarrhea among children under two years of age in rural Bangladesh. Point-of-use water treatment with chlorine and safe storage have been shown to effectively improve water quality and reduce childhood diarrhea in various settings [29,30]. We hypothesized that children under two who drink treated and safely stored tubewell water would have less diarrhea than those who drink untreated tubewell water stored with the standard water handling practices of rural Bangladesh. We also hypothesized that, while safe storage would be beneficial compared to standard practice, it would lead to larger reductions in diarrhea when combined with chlorination (compared to safe storage alone).

## Methods

### Ethics

The protocol for this trial and supporting CONSORT checklist are available as supporting information; see S1 Protocol and S1 CONSORT Checklist. The study protocol was reviewed and approved by institutional review boards at the University of California, Berkeley, Centers for Disease Control and Prevention (CDC) and the International Centre for Diarrhoeal Disease Research, Bangladesh (icddr,b). The original protocol included a blinded fourth study arm intended to receive placebo tablets without the active chlorine disinfectant to allow assessment of courtesy bias and placebo effects. However, the use of a placebo was not approved by the local institutional review board on ethical grounds and was not implemented. The inclusion of a control arm in the study was considered ethically justified by both the study investigators and the human subject review committees as this group followed the everyday water handling practices that are the norm in rural Bangladesh. All participants provided written informed consent prior to enrollment. The study was registered at ClinicalTrials.gov (NCT01350063).

### Participant Selection and Enrollment

We selected the study location based on local groundwater chemistry. Groundwater in parts of Bangladesh is rich in iron, which exerts chlorine demand and limits the free chlorine residual available for pathogen inactivation. We therefore conducted the study in Mymensingh district in central Bangladesh where iron concentrations in groundwater are low (S1 Fig.) [26]. A pilot study in the area confirmed low iron presence in tubewell water, allowing consistent free chlorine residual within the range recommended by the World Health Organization (WHO) and the CDC.

We selected 87 villages in the Fulbaria sub-district of Mymensingh; these were randomly selected from a total of 106 villages in the sub-district that remained available after excluding areas where pilot activities had taken place. We screened the selected villages for households that consistently relied on a shallow tubewell (<250 ft) as their primary source of drinking water, had no complaints of iron presence in their tubewell, had a child between the ages of six and 18 months living in the household (index child), and did not plan to move within the study period. Families with iron complaints were excluded as an initial chlorine dosing exercise in the study area indicated self-reported iron to be a sensitive predictor of whether a household’s tubewell water would fall short of acceptable chlorine residual (S1 and S2 Tables). The lower age limit of six months was chosen because of the national Bangladeshi policy that stipulates that infants under six months should be exclusively breastfed and not given any water. The upper age limit of 18 months was chosen to ensure that the majority of the children would be under the age of two during the follow-up period, and would represent the age group that is most vulnerable to waterborne illness and its longterm sequalae [31]. However, once a household was enrolled, all children between the ages of six months and five years living in the household were considered eligible for data collection.

We selected a random subset of 1800 households from the 2515 households that met our eligibility criteria. Households in Bangladesh are typically clustered into compounds consisting of extended families. If there were multiple eligible households in a compound, only one household was randomly selected to avoid correlated diarrhea outcomes among households in the same compound. In households with more than one child in the eligible age range (six months to five years), all eligible children were enrolled. Field staff approached selected households to obtain informed consent from the primary caregiver of children under the age of two in the household and administer a baseline questionnaire that assessed the pre-intervention child health status, water and sanitation practices, demographics and socioeconomic status.

### Randomization Assignment and Allocation Concealment

The lead investigators (AE and AMN) generated the randomization sequence using the random allocation function of STATA software (version 10.1, STATA Corp., College Station, TX). The study area was divided into 15 distinct geographical regions; in each region eligible households were listed in the order they were identified during screening, and block randomization with a block size of three was applied to assign 1800 households to one of three study arms: (1) chlorine plus safe storage: sodium dichloroisocyanurate (NaDCC) tablets and a narrow-mouth vessel with a lid and tap; (2) safe storage: a narrow-mouth vessel with a lid and tap; and (3) control: no intervention. Field teams delivering the interventions and collecting follow-up data were informed about the randomization assignment after the completion of participant enrollment and baseline data collection.

### Intervention Delivery and Promotion

NaDCC tablets have proven effective in improving water quality in other settings [21,32] and been found acceptable to users in a low-income urban community in Dhaka, Bangladesh [21]. The tablets are easier to store, handle and correctly dose than liquid forms of chlorine [33]. A pilot exercise was conducted in the study area to identify the ideal dose that provides a minimum free chlorine residual of 0.2 mg/L to ensure adequate disinfection and a maximum residual of 2 mg/L to minimize taste and odor concerns, as recommended by the CDC. One 33 mg NaDCC tablet in 10 liters of water, corresponding to an initial free chlorine dose of 2 mg/L, was identified as adequate (S3 Table). We identified a commercial water storage jar with a tightly fitting lid, a narrow mouth (10.5 cm diameter) and a durable tap as a suitable safe storage container (S2 Fig.).

Field staff distributed the intervention products to study households following the completion of baseline data collection in all households (September 2011) and demonstrated their use, including how to clean the safe storage container with a provided brush and detergent. They left an illustrated instruction sheet at a visible spot in the household to serve as a reminder. They instructed participants in both intervention arms to discard any remaining water after 24 hours and collect a fresh 10-liter batch, and to exclusively give treated and/or safely stored water to all children under five that live in the household. The field team continued to visit households approximately once a month for one year (October 2011 to November 2012) to promote correct and consistent use of the products and replenish the supply of tablets. In order to prevent potential differential Hawthorne effects between study arms, where Hawthorne effect is defined as subjects perceiving or reporting spurious health benefits as a consequence of “being watched and unusual attention being paid” [34,35], the control group was visited with the same frequency as the intervention groups. The promotion activities in this group provided no information on water treatment or safe storage but focused on general information on diarrhea and oral rehydration therapy, which is not expected to affect diarrhea prevalence in the control group. Households in the control group were given the same safe storage container as the intervention arms upon completion of the study. Additionally, the field team provided oral rehydration solution to participants from all three arms upon request throughout the study.

### Outcome Definition and Measurement

A separate field team conducted unannounced monthly follow-up visits for one year (October 2011 to November 2012), on average two weeks after each promotion visit, to record caregiver-reported two-day and seven-day prevalence of diarrhea (defined as three or more loose stools within a 24-hour period) in index children and any additional children under five that live in the enrolled household. We specified *a priori* to use seven-day prevalence in our analysis unless we detected evidence of differential recall bias (i.e., difference in the magnitude of effect estimates obtained using two- vs. seven-day recall) [36]. In addition to diarrhea, the field team recorded caregiver-reported prevalence of skin rashes and ear infections to serve as negative control outcomes [37]; these were symptoms that could not plausibly be affected by the drinking water interventions and were used to detect potential differential reporting bias associated with subjective, self-reported outcomes in response to non-blinded interventions. To minimize bias during data collection, the field team conducting the follow-up visits was not informed about which symptoms were study outcomes of interest versus negative control outcomes.

We originally designed the trial to have five follow-up visits, which largely overlapped with the dry season (October 2011 through May 2012) in Bangladesh. We hypothesized *a priori* that the impact of the drinking water interventions on child diarrhea would vary by season; we therefore extended the study by five additional visits beyond the original design to capture data during the rainy season (June 2012 through November 2012).

During each follow-up visit, the team monitored intervention uptake by recording self-reported use, conducting spot checks on the presence and status of the intervention products and collecting stored water samples in all households (n = 600) in the chlorine arm to test for free chlorine residual. During one follow-up round, samples for chlorine testing were also collected in a subset of households (n = 15) in the safe storage arm randomly selected from among those located closest to households in the chlorine arm to assess any spillovers. In a rotating systematic subsample of 10% of households in all study arms, the field team also collected tubewell and stored water samples for microbiological testing; only one type of sample was collected if the other type was not available (e.g., storage vessel empty at time of interview) in the household selected for systematic sampling. Samples were transported on ice to the field laboratory. Laboratory staff measured free chlorine residual with the n,n-diethyl-p-phenylenediamine (DPD) colorimetric method using a digital colorimeter (Hach, Loveland, CO, USA; lower estimated detection limit: 0.02 mg/L; precision ± 0.05 mg/L). *E*. *coli* was enumerated with membrane filtration using U.S. EPA Method 1604 within eight hours of sample collection [38]. Quality control measures including 10% blanks and 10% duplicates were followed. *E*. *coli* concentration was measured in colony forming units (CFU) per 100 mL, and samples were classified according to the WHO thresholds of no risk (<1 CFU/100 mL), low risk (1–10 CFU/100 mL), moderate risk (11–100 CFU/100 mL) and high risk or above (>100 CFU/100 ml) [39].

### Statistical Methods

Our primary outcome was the seven-day period prevalence of caregiver-reported diarrhea in index children (6–18 mo at enrollment). We conservatively sized the study to detect a difference in the two-day prevalence of diarrhea due to safe storage plus chlorination over safe storage alone. We assumed 14% two-day diarrhea prevalence in the control group based on data from a large-scale study in rural Bangladesh (SHEWA-B) [40], 11.6% prevalence in the safe storage group based on 30% diarrhea reduction due to safe storage [30] and 55% of participants storing water in the home [12], and 9.1% prevalence in the combined intervention group based on 35% diarrhea reduction due to safe storage plus chlorination [29]. Assuming one child of eligible age per household, an intra-cluster correlation coefficient (ICC) of 0.13 for repeated observations within a child based on the SHEWA-B study, 5% drop-out and a one-sided α of 0.05, we calculated that 575 participants visited five times would provide 84% power to detect the difference between 11.6% and 9.1% diarrhea prevalence. We enrolled 600 households in each study arm; we conducted five visits during the dry season and five additional visits during the monsoon season to ensure sufficient power to individually detect a health difference in either season.

We conducted all statistical analyses using STATA software (version 12.1, STATA Corp., College Station, TX). We calculated disease prevalence ratios (PR) between pairs of study arms using generalized linear models with a log link, a binomial error distribution, and robust standard errors to account for clustering due to longitudinal sampling and multiple children per household when there was more than one eligible child [41]. In the case of loss to follow-up, all observed data for a given child prior to leaving the study were used in the analysis. We investigated effect modification by two pre-specified characteristics by including interaction terms in the regression models: season (dry vs. monsoon) and child age (6–12 mo, 13–18 mo and > 18 mo at enrollment). We calculated the ICC for repeated measures within children using one-way ANOVA analysis with the loneway function in STATA.

Our secondary outcome was fecal contamination of stored water, defined as the proportion of samples with an *E*. *coli* count exceeding the WHO thresholds of no risk, low risk and moderate risk. We compared stored as well as source water quality across study arms using chi square tests (or Fisher’s exact test in the case of sparse data) for the proportion of samples in these risk categories and conducted subgroup analyses with season.

All analyses were conducted by the original assigned groups in an intention-to-treat analysis. The complete data management process and statistical analyses for the primary and secondary outcomes were independently replicated by two investigators (AE and AMN) to ensure identical, replicable results. Investigators had no access to outcome data until field activities were complete. CONSORT guidelines were followed [42].

## Results

### Baseline Characteristics

Baseline data collection was conducted between July and September 2011. Households in the three study arms had similar distributions of demographics, socioeconomic status and water, sanitation and hygiene-related practices at baseline (Table 1).

**Table 1:** Summary of baseline characteristics by study group.

	Control		Safe storage		Chlorine + safe storage	
	(N = 600 HHs)		(N = 600 HHs)		(N = 600 HHs)	
	N	Mean/%	N	Mean/%	N	Mean/%
**Demographics and socioeconomics**						
Number of index children 6–18 mo at enrollment	605		603		606	
Number of siblings 19–60 mo at enrollment	133		130		133	
Mean age of respondent (years)	584	26	587	26	587	25
Mean number of persons per HH	584	5.3	587	5.4	586	5.3
Mean monthly HH income (USD)	573	92	583	95	582	93
Mean number of rooms in HH	584	1.6	587	1.6	587	1.6
Mean land owned by HH (acres)	578	0.5	584	0.5	582	0.5
% of HHs with:						
*Kaccha* walls ^a^	584	34	587	35	587	36
Electricity	584	34	587	36	586	35
Cell phone	584	68	587	67	586	68
TV	584	22	587	22	586	19
% of mothers with 0 yrs of education	584	28	587	27	587	27
**Water, sanitation and hygiene practices**						
% of HHs with drinking water obtained:						
Directly from tubewell	582	41	587	40	586	43
From narrow-mouth container ^b^	582	45	587	44	586	42
From wide-mouth container	582	13	587	14	586	15
% of HHs that treat drinking water	584	2	587	2	587	1
% of HHs with:						
Improved sanitation facility ^c^	584	32	587	37	587	33
Unimproved sanitation facility ^d^	584	51	587	47	587	47
No sanitation facility	584	17	587	16	587	19
% of HHs where children <2 yrs defecate:						
In latrine, potty or cloth	584	24	587	26	587	28
In courtyard or living area	584	96	587	94	587	94
Outside compound area	584	4	587	7	587	6
% of HHs with:						
Handwashing station (HWS)	584	80	587	81	586	81
HWS <10 steps from latrine	584	31	587	34	586	33
HWS with water	584	72	587	72	586	72
HWS with soap	584	32	587	36	586	34
**Health indicators in index children (6–18 mo at enrollment)**						
Two-day % prevalence of:						
Diarrhea	605	11	603	11	606	9
Skin rash	605	14	602	15	606	15
Ear infection	605	4	602	4	604	6
Seven-day % prevalence of:						
Diarrhea	605	16	603	16	606	14
Skin rash	605	16	602	17	606	16
Ear infection	605	5	602	5	604	7

HH: Household; USD: US dollars; HWS: Handwashing station

^a^ *Kaccha* walls refer to natural wall materials including jute, bamboo and mud.

^b^ The narrow-mouth containers used by all 3 groups were almost exclusively *kolshis*, which have a narrow mouth but a wide brim and no lid, allowing contamination.

^c^ Improved facilities include flush/pour flush latrines that drain to piped sewer, septic tank, or off-set pit; pit latrines with slab and water seal or with slab, no water seal but lid; and composting toilets.

^d^ Unimproved facilities include flush/pour flush latrines that drain into the environment; open pits; pit latrines without slab; pit latrines with slab but no water seal and no lid; and hanging toilets.

Water treatment was rarely practiced among study participants at baseline, with 1–2% of households reporting treating their drinking water (Table 1). Of the 30 total households that reported water treatment, 26 boiled, two used a cloth filter and boiled, one used chlorine tablets and one used a commercial filter. In 40–43% of households, respondents retrieved water directly from the tubewell when asked to provide a glass of water as if giving it to their young children; the remainder obtained water from storage containers (Table 1). The most frequently observed water storage containers were *kolshis* and jugs.

### Longitudinal Follow-Up

**Fig 1:**
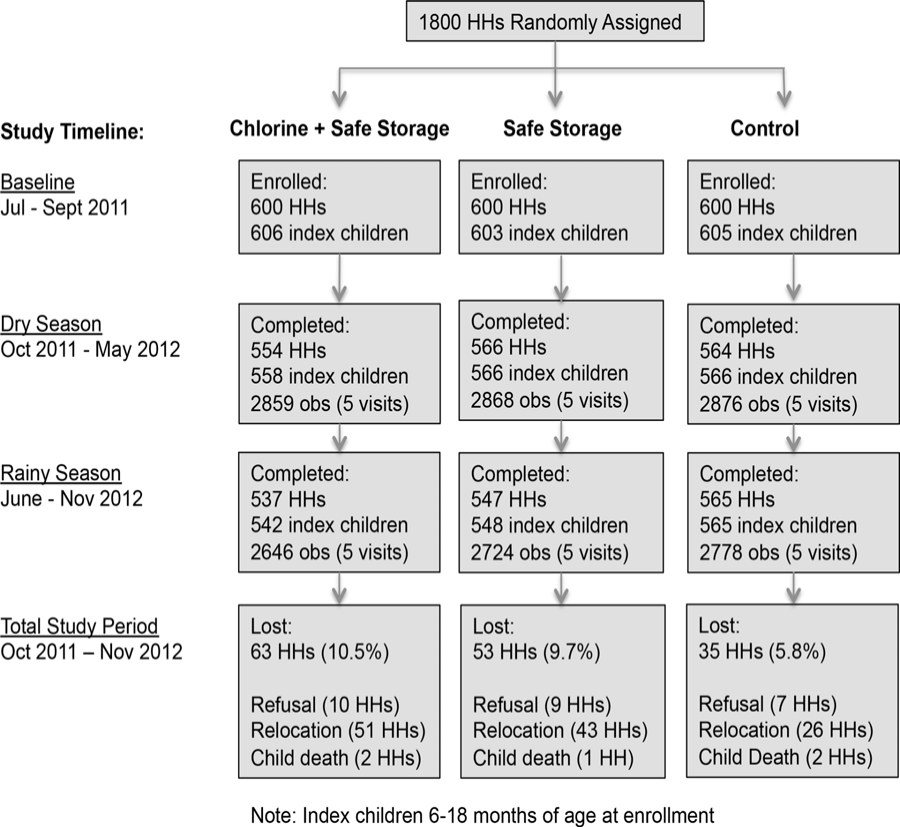
Flowchart of study participation.

Of the 1800 households enrolled and randomly assigned into study arms, 1786 received promotion visits by the field team (including control households that received visits unrelated to safe water); 14 households were lost due to relocation (n = 10), refusal to participate (n = 3) and death of enrolled child (n = 1) before the onset of promotion activities and the delivery of hardware to intervention households. A total of 10 follow-up visits per household were conducted between October 2011 and November 2012; 1649 households completed the study while a cumulative 151 households were lost to follow-up due to relocation (n = 120), refusal to participate (n = 26), and death of enrolled child (n = 5) (Fig. 1). The refusal rate was similar between study arms (10 households in chlorine plus safe storage arm, 9 households in safe storage arm and 7 households in control arm), suggesting no difference in willingness to participate. Households that left the study were similar in their characteristics to households that completed the study, and the balance of baseline variables between the three study arms was maintained among the households that remained in the study (S4 and S5 Tables), suggesting that loss to follow-up did not depend on covariates; we assumed that data were missing completely at random [43].

### Intervention Uptake

The safe storage and chlorination interventions achieved high uptake during the one-year study period. The delivered storage container was observed to contain water in 91% of spot check observations in the safe storage arm and 87% of observations in the chlorine arm over all follow-up visits (Table 2). Of the households in the chlorine arm that had water in the intervention container at the time of the visit, 83% had free chlorine residual over the minimum CDC-recommended value of 0.2 mg/L (Table 2); the percentage of households in compliance with this target was stable over the study period (S3 Fig.). None of the tested households in the safe storage arm had a free chlorine residual detectable at the 0.2 mg/L threshold. When asked to provide a glass of water for their young children, 84–89% of caregivers in the intervention arms retrieved water from the provided container (Table 2). In control households, 70% of respondents retrieved water from a storage container; among these, the most commonly observed containers were *kolshis* (64%) and jugs (33%). Only 2% of control households reported treating their drinking water and boiling was the predominant method, suggesting that control households continued to follow their baseline water handling practices during the follow-up period.

**Table 2:** Uptake indicators in intervention groups (cumulative data from 10 follow-up visits).

	Safe storage		Chlorine + safe storage	
	N	%	N	%
Water use from intervention container:				
Observed to retrieve water for children from elsewhere	5613	11	5496	16
Reported to give index child water from elsewhere ^a^	5592	14	5473	18
Reported to give older sibling water from elsewhere ^b^	1209	22	1222	32
Observed status of intervention container:				
Container not present	5613	5	5496	7
Container empty	5613	4	5496	6
Container full but uncovered	5613	1	5496	1
Container full and covered	5613	90	5496	86
Reported to fill intervention container:				
On day of interview	5613	25	5496	40
Day before interview	5613	66	5496	49
Two or more days before interview	5613	8	5496	12
Reported to add chlorine tablets to intervention container:				
On day of interview	—	—	5496	40
Day before interview	—	—	5496	47
Two or more days before interview	—	—	5496	13
Reported having chlorinated water available:	—	—	5496	87
Free chlorine residual in intervention container:		—		
No sample available	—	—	5496	14
Residual <0.2 mg/L	—	—	5496	15
Residual 0.2–2 mg/L	—	—	5496	66
Residual 2–5 mg/L			5496	4
Residual >5 mg/L	—	—	5496	1

^a^ Index children 6–18 mo of age at enrollment.

^b^ Older siblings 19–60 mo of age at enrollment.

### Water Quality

**Fig 2:**
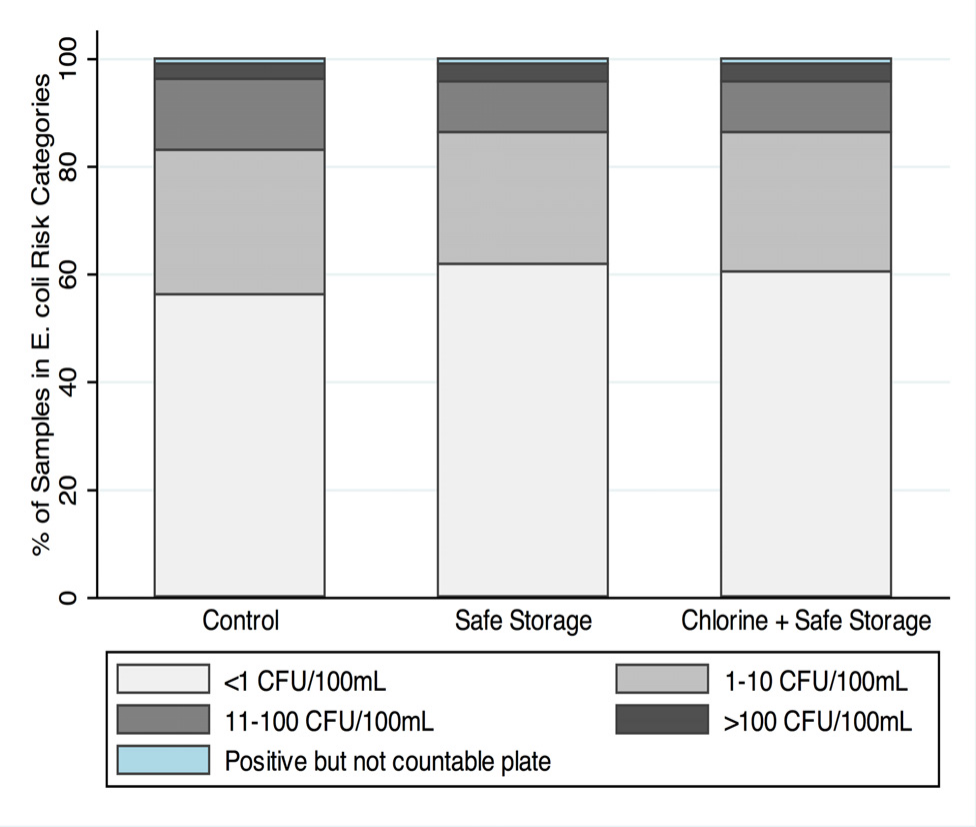
Categories of *E*. *coli* counts in tubewell water across arms (see S6 Table).

The field team collected 1726 source water samples and 1676 coupled stored water samples over the study period. Among tubewell samples, 41% were positive for *E*. *coli*. In 14% of samples, *E*. *coli* counts were over the low-risk limit of 10 CFU/100 mL and 3% exceeded the moderate-risk limit of 100 CFU/100 mL (Fig. 2); there were no differences in the percentage of samples falling in these risk categories between any pairs of study arms (p>0.05), indicating similar source water quality across study arms (S6 Table). Contamination of tubewell water was more common during the monsoon season than in the dry season (S4 and S5 Figs, S6 Table), with a higher percentage of samples positive for *E*. *coli* (χ^2^ p-value<0.005) and higher log10 *E*. *coli* counts (Wilcoxon rank-sum test p-value<0.005).

**Fig 3:**
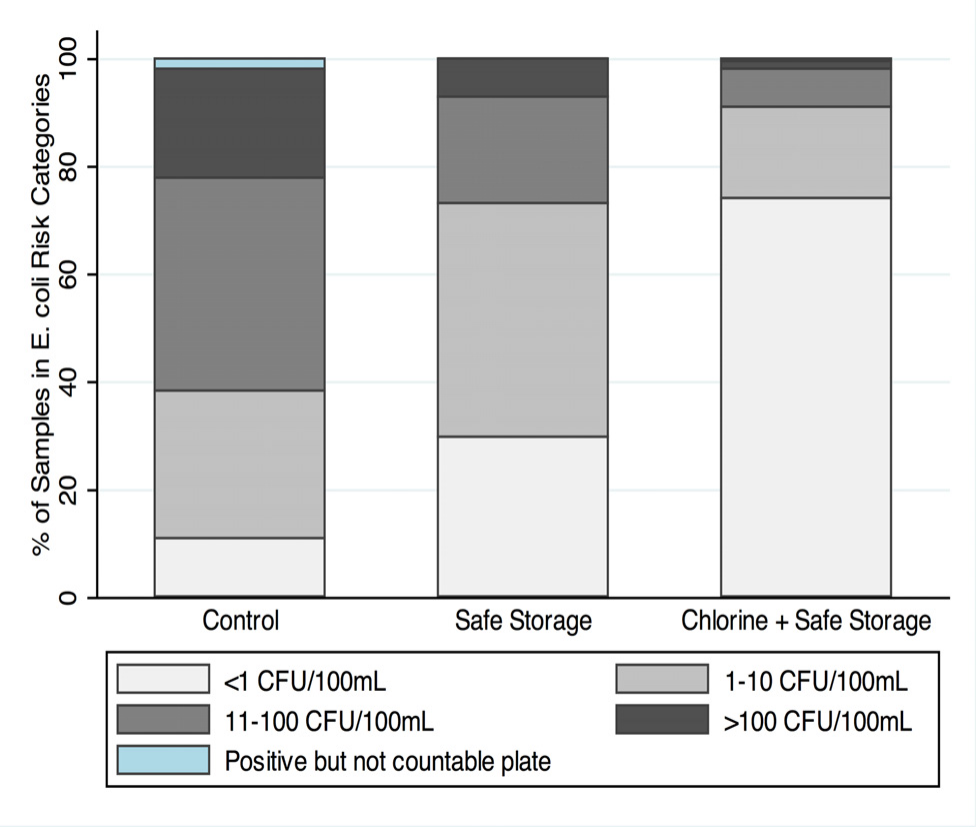
Categories of *E*. *coli* counts in stored water across arms (see S6 Table).

**Fig 4:**
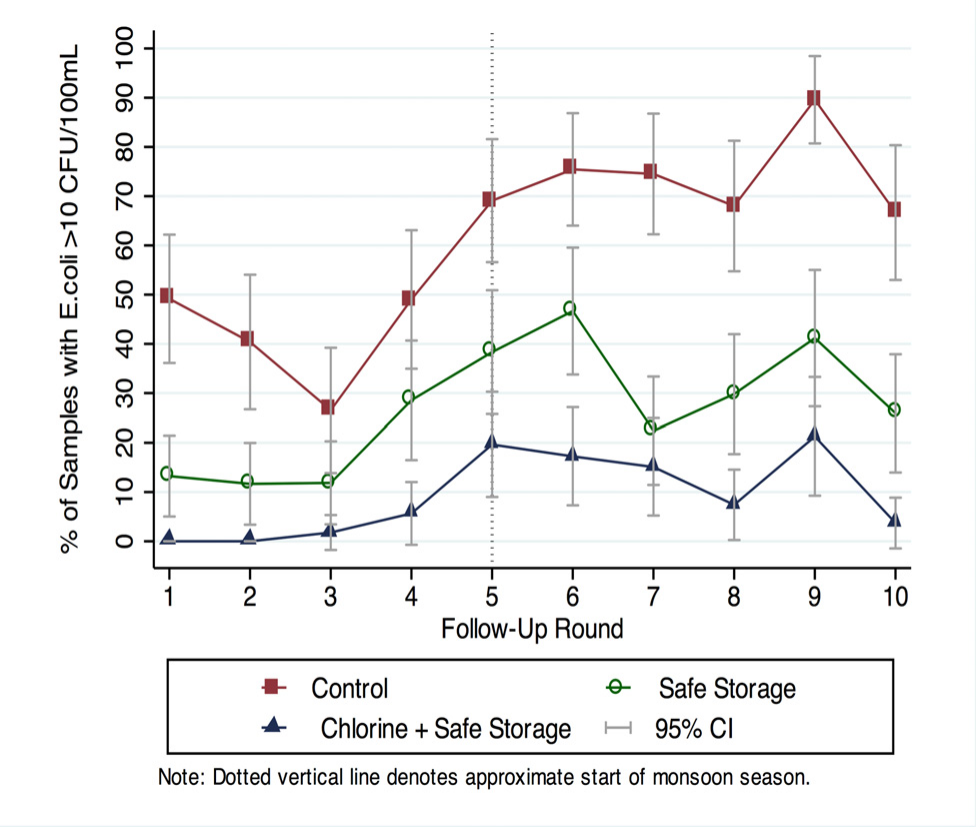
Temporal trend in percentage of stored water samples with *E*. *coli* >10 CFU/100 mL.

**Fig 5:**
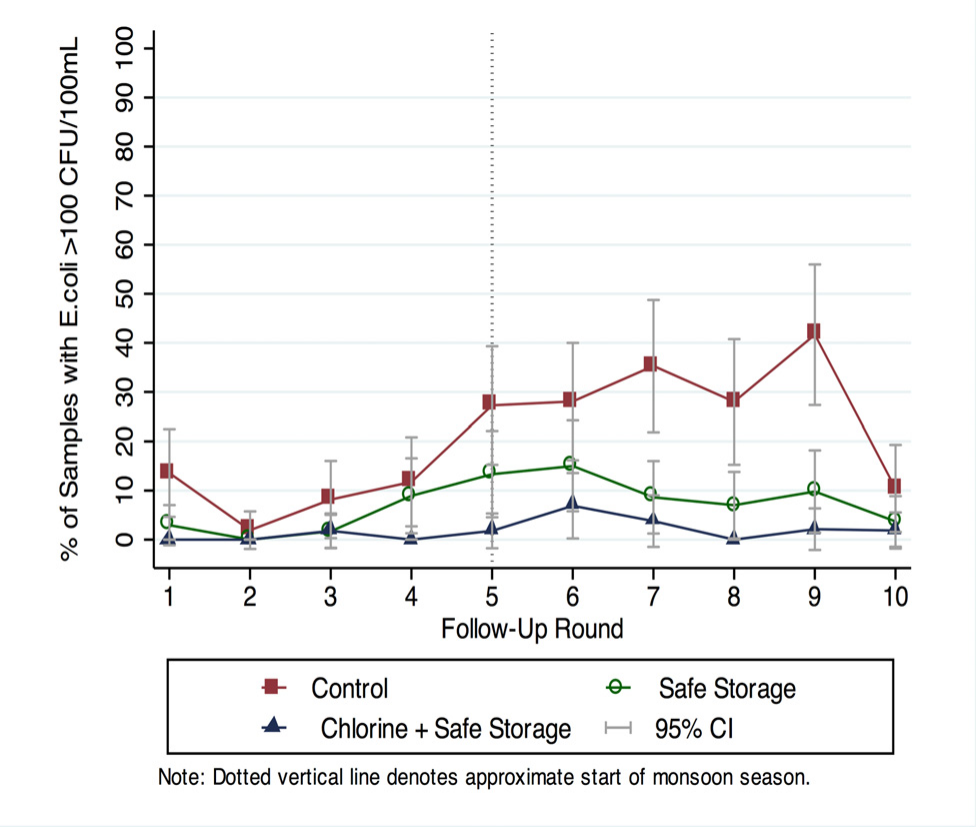
Temporal trend in percentage of stored water samples with *E*. *coli* >100 CFU/100 mL.

Stored water quality showed marked differences between the three arms. In the control arm, 89% of samples were positive for *E*. *coli*, suggesting widespread contamination during household storage, compared to 70% in the safe storage arm and 26% in the combined safe storage and chlorination arm (Fig. 3). The percentage of samples with *E*. *coli* >10 CFU/100 mL was 61% in the control arm, 27% in the safe storage arm and 9% in the chlorine arm, and the percentage of samples with *E*. *coli* >100 CFU/100 mL was 21% in the control arm, 7% in the safe storage arm and 2% in the chlorine arm (Fig. 3). All differences were significant between each pair of study arms (p<0.05) (S6 Table). The proportion of households that had higher *E*. *coli* counts in their stored water than their source water was 77% in the control arm, 56% in the safe storage arm, 38% in the chlorine arm with suboptimal (<0.2 mg/L) chlorine, 14% in the chlorine arm with optimal (≥0.2 mg/L) chlorine. Stored water contamination was more pronounced during the monsoon season compared to the dry season across all three study arms (Figs. 4 and 5, S6 Table), reflected in a higher percentage of *E*. *coli* positive samples (χ^2^ p-value<0.005) and higher log10 *E*. *coli* counts (Wilcoxon rank-sum test p-value<0.005).

### Child Diarrhea

**Fig 6:**
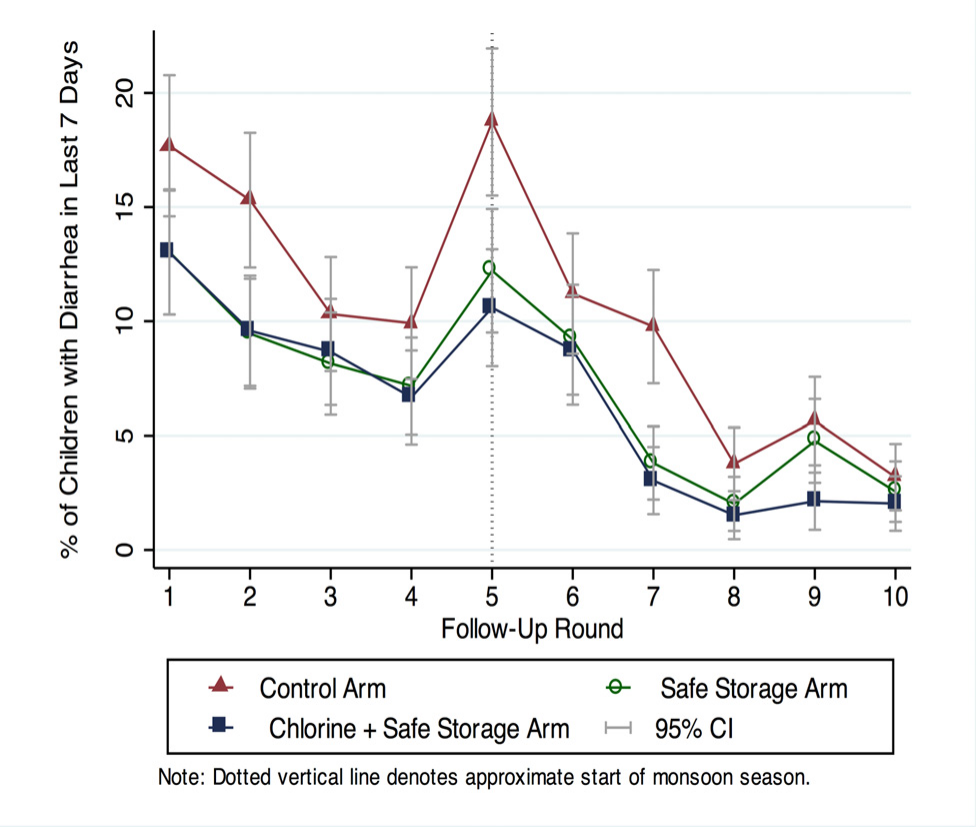
Temporal trend in diarrhea prevalence in children 8–32 months of age.

A total of 1814 index children were enrolled into the study. Mean index child age over the follow-up period was 20 months (range: 8–32 months). Diarrhea prevalence in index children in the control arm was 10.6% over the study period; prevalence decreased with increasing study duration in all three arms but showed a peak at the onset of the monsoon (Fig. 6). The youngest age group had the highest diarrhea prevalence (Table 3). The ICC for repeated diarrhea measures within children was 0.06.

**Table 3:** Prevalence of diarrhea across study arms (7-day recall period).

	Control		Safe storage						Chlorine + safe storage									
	N	Prev %	N	Prev %	PR ^a^	95% CI		p ^c^	N	Prev %	PR ^a^	95% CI		p ^c^	PR ^b^	95% CI		p ^c^
**Main analysis (among children 8–32 mo of age) ^d^**																		
All	5654	10.6	5592	7.3	0.69	(0.60,	0.80)		5505	6.7	0.64	(0.55,	0.73)	—	0.92	(0.79,	1.08)	—
**Interaction with season (among children 8–32 mo of age) ^d^**																		
Dry season (ref)	2876	14.5	2868	10.0	0.70	(0.59,	0.82)	—	2859	9.7	0.68	(0.57,	0.79)	—	0.97	(0.82,	1.16)	—
Wet season	2778	6.7	2724	4.5	0.67	(0.52,	0.85)	0.77	2646	3.5	0.52	(0.40,	0.68)	0.12	0.78	(0.59,	1.04)	0.23
**Interaction with child age at enrollment**																		
06–12 mo (ref)	3415	11.7	3541	7.9	0.67	(0.56,	0.80)	—	3410	7.2	0.61	(0.51,	0.73)	—	0.91	(0.75,	1.10)	—
13–18 mo	2239	9.0	2051	6.4	0.71	(0.55,	0.91)	0.75	2095	6.1	0.67	(0.53,	0.86)	0.56	0.95	(0.73,	1.24)	0.81
19–60 mo	1169	4.4	1180	3.7	0.84	(0.49,	1.43)	0.42	1182	3.5	0.78	(0.43,	1.40)	0.43	0.93	(0.50,	1.73)	0.95

^a^ Prevalence ratio refers to comparison against control group.

^b^ Prevalence ratio refers to comparison against safe storage group.

^c^ p-value for interaction term against reference category.

^d^ Index child 8–32 mo of age during follow-up (6–18 mo at enrollment).

Compared to the control arm, caregiver-reported diarrhea in index children was significantly reduced in both the chlorine plus safe storage arm (PR = 0.64, 0.55–0.73) and the safe storage arm (PR = 0.69, 0.60–0.80); there was no difference in the chlorine plus safe storage versus safe storage arm (PR = 0.92, 0.79–1.08) (Table 3). Controlling for use of drinking water from other sources for children (16% in chlorine plus safe storage arm vs. 11% in safe storage arm, Table 2) did not change the prevalence ratio between the two intervention arms (PR = 0.92, 0.79–1.08). Prevalence ratio estimates using two-day prevalence were similar, consistent with no differential recall by study group (S7 Table). There was no significant difference in the seven-day prevalence of the negative control outcomes in the chlorine plus safe storage arm (skin rash PR = 0.86, 0.66–1.10, ear infection PR = 0.94, 0.61–1.45) or the safe storage arm (skin rash PR = 0.88, 0.68–1.14, ear infection PR = 1.26, 0.84–1.91) compared to control (S8 Table). There appeared to be increased protection from safe storage and chlorination combined in the monsoon season compared to the dry season but the interaction was not significant (Table 3). There was no evidence of differential intervention impact by child age for either intervention (Table 3).

## Discussion

### Summary of Findings

The interventions achieved high uptake in the study population. We found significant improvements in stored water quality due to safe storage with and without chlorination, with 7% of stored water samples in the safe storage arm and 2% of stored water samples in the chlorine arm exceeding moderate-risk contamination levels compared to 21% in controls (Fig. 3). There was 31% reduction in diarrhea prevalence in children 8 to 32 months old in the safe storage arm (PR = 0.69, 0.60–0.80) and 36% reduction in the combined safe storage and chlorination arm (PR = 0.64, 0.55–0.73) compared to controls, with no difference between the two intervention arms (PR = 0.92, 0.79–1.08) (Table 3).

### Effectiveness of Safe Storage vs. Safe Storage and Chlorination

Our findings indicate that safe storage, alone or combined with chlorination, was effective in reducing child diarrhea in rural Bangladesh compared to standard practice, and, given safe storage, there was no additional benefit from chlorination. There are several possible explanations for this. It is possible that, if drinking water is safely stored and handled, there truly is no added benefit from adding chlorine in this particular setting. Our water quality testing results support this explanation; the source water quality was relatively good in the study area, and contamination of water stored in households was common, as evidenced by the high percentage of *E*. *coli* positive stored water samples in the control group (Figs. 2 and 3). Safe storage combined with chlorination was more effective at preventing contamination (especially low-level contamination) of stored water than safe storage alone but both achieved marked protection against moderate and high levels of point-of-use contamination compared to the control arm (Figs. 4 and 5); 2% of samples in the safe storage plus chlorination arm and 7% of samples in the safe storage arm had *E*. *coli* exceeding 100 CFU/100 mL as opposed to 21% in the control arm (S6 Table). In this context, the equivalent reduction in diarrhea in both intervention arms might suggest that safe storage alone sufficiently reduced waterborne pathogen transmission. The magnitude of the reduction was consistent with diarrhea reduction associated with safe storage in another setting [30].

It is also possible that the lack of additional diarrhea reduction from chlorination in addition to safe storage may be due to the presence of chlorine-resistant organisms in traditionally stored groundwater in the study setting. While chlorine is very efficacious in inactivating bacterial pathogens, its efficacy is only moderate against viruses and poor against protozoan cysts [2,44]. If groundwater stored with standard practice in rural Bangladesh becomes predominantly contaminated with chlorine-resistant diarrheagenic pathogens due to contact from infected household members, safe storage would be expected to reduce diarrhea by reducing contact but chlorination would not provide additional protection against the pathogens that enter the safe storage container. This type of resistant fecal contamination in chlorinated stored water would not be detected by our *E*. *coli* measurements as *E*. *coli* is effectively inactivated by chlorine and is not a good indicator for more resistant organisms such as *Giardia* or *Cryptosporidium*. However, bacterial pathogens including enterotoxigenic *E*. *coli*, *Shigella*, *Campylobacter jejuni* and *Vibrio cholerae* are frequently isolated in stool samples from children with diarrhea in Bangladesh [45–47]. It is therefore unlikely that the dominance of chlorine-resistant pathogens in stored drinking water can explain the lack of additional diarrhea reduction from safe storage plus chlorination compared to safe storage alone.

Aversion to the use of chlorine could have been another factor behind the lack of additional diarrhea reduction from chlorination if the user uptake of the NaDCC tablets had been poor among study participants. However, stored water samples from households in the chlorine plus safe storage arm consistently showed acceptable levels of free chlorine residual. Moreover, the large majority of participants in both intervention arms were observed to retrieve water from the provided storage container when asked to provide a glass of water as if giving it to their children, and controlling for the use of water from other sources did not change the diarrhea prevalence ratio between the two intervention arms.

### Limitations

One limitation of our study is that it employed a non-blinded design with self-reported outcomes. An alternative explanation for the similar health impact in the two intervention groups may therefore be that the reported reductions in diarrhea in both groups may be a result of courtesy bias and/or a placebo effect due to provision of intervention products to participants. It has been suggested that exaggerated reporting of health improvements in non-blinded studies with self-reported outcomes can partially or fully explain health benefits documented in previous water treatment trials [48,49]; indeed, such effects would not be additive for combined interventions.

Previous blinded studies on household water treatment have shown no diarrhea reduction from chlorination [32,50–52]. While these studies mitigated reporting bias by including a placebo arm, other factors such as small sample size [52], and low intervention uptake and low diarrhea prevalence in the study population [51] make their interpretation difficult. Our study enrolled 1,800 households in a setting with 10% diarrhea prevalence in children <2 years and achieved exceptionally high uptake of both the chlorine and safe storage interventions. Our findings are consistent with the blinded chlorination study by Jain et al. where both the chlorine and placebo groups were given safe storage containers as part of the intervention; the authors found improved water quality in both groups and no difference in diarrhea, suggesting that the protection provided by the safe storage containers might have sufficiently reduced diarrhea in both groups [32].

Few studies have focused on safe storage as a standalone intervention; these have found improvements in point-of-use water quality and/or reductions in diarrheal disease [30,53,54]. While blinding is a desired study feature to minimize reporting bias, a safe storage intervention is nearly impossible to blind. However, we implemented several measures to minimize biased reporting. The interventions were distributed and promoted by different field staff than those who collected the health data to minimize courtesy bias. We collected negative control outcomes including skin diseases and ear infections that would not be improved by the interventions; we found no impact of either intervention on these caregiver-reported symptoms, suggesting no evidence of a placebo effect. Finally, our stored water quality measurements present an objective intermediate outcome on the causal chain between the interventions and child health, and provide support for a diarrhea reduction of similar magnitude in both intervention arms.

We conducted our study in a setting with low groundwater iron concentration to maximize the effectiveness of our chlorine intervention, which is reflected in the consistently high residual free chlorine concentrations we observed in participants’ stored water. Our finding that chlorination led to no additional diarrhea reduction beyond safe storage alone is expected to be generalizable to other areas of Bangladesh where higher levels of iron would further limit the effectiveness of chlorine as a disinfectant. Our study region also had low groundwater arsenic concentrations [26]. Assuming an inverse relationship between arsenic concentration and microbiological contamination as previous research suggests [11, 15], we expect that the added benefit of chlorination (given safe storage) would be even smaller in high-arsenic regions with presumably better microbiological groundwater quality. On the other hand, safely storing drinking water is expected to effectively reduce child diarrhea in such settings as the benefits from minimizing point-of-use contamination would be more pronounced when point-of-source contamination is low [23]. We expect our findings on safe storage to be generalizable to a variety of settings where the microbiological quality of the source water is relatively good and storing drinking water prior to consumption is common practice.

Finally, it is important to note that this study was an efficacy trial where our objective was to find out whether consistently treating and safely storing tubewell water would improve water quality and reduce diarrhea in rural Bangladesh in order to assess the magnitude of the diarrheal disease burden associated with consuming shallow tubewell drinking water. We identified easy-to-use, aspirational products, provided them to participants for free and promoted their use through regular household visits. While both products achieved high uptake among study participants and reduced diarrhea, their uptake and consequently their health impact is likely to be lower in the absence of free provision and intensive promotion efforts. However, an important public health implication of this study is that, in settings with relatively good source water quality, safe storage is sufficient to markedly reduce waterborne illness, eliminating the need for continuous purchase or maintenance of water treatment products. The economic and behavior change burden associated with the repeated purchase of consumable supplies such as chlorine can be a barrier against the adoption of safe water technologies in resource-poor settings, where users express a preference for durable products [55]. Low user adoption in turn is one of the key limiting factors for the health impact of water quality interventions [56]. Making a one-time investment in a safe storage container might require more modest behavior change than using water treatment products that need to be periodically replenished, allowing higher uptake and a larger health impact.

### Conclusions

Safe storage, used alone or in combination with chlorination, markedly improved the microbiological quality of stored water in the study setting. Both interventions significantly reduced diarrhea prevalence in young children. Chlorination, however, did not provide an additional reduction beyond that seen with safe storage. This is plausible because the level of microbiological contamination was low at the water source, and both safe storage alone and safe storage combined with chlorination reduced high-level contamination of stored drinking water compared to the control arm. Our findings indicate that unsafe handling during storage in the household is the dominant mechanism for contamination of tubewell drinking water and that safe storage as an effective and readily adopted standalone intervention could substantially reduce waterborne illness among young children in rural Bangladesh. Efforts should be undertaken to implement and evaluate long-term efforts for safe water storage in Bangladesh.

## Supporting Information

S1 CONSORT checklist CONSORT Checklist. (DOCX)

S1 Fig Iron map of Bangladesh [26]. (TIF)

S2 Fig Safe storage container with tightly fitting lid, narrow mouth and tap. (TIF)

S3 Fig Temporal trend in percentage of households with chlorine residual ≥0.2 mg/L. (TIF)

S4 Fig Temporal trend in percentage of source water samples with *E*. *coli* >10 CFU/100 mL. (TIF)

S5 Fig Temporal trend in percentage of source water samples with *E*. *coli* >100 CFU/100 mL. (TIF)

S1 Table Self-reported iron versus free chlorine residual among 52 wells at 30 min after chlorination. (DOCX)

S2 Table Sensitivity, specificity, positive predictive value (PPV) and negative predictive value (NPV) of self-reported iron complaints as predictor of free chlorine residual <0.2 mg/L vs. ≥0.2 mg/L among 52 wells at 30 min after chlorination. (DOCX)

S3 Table Free chlorine residual among 52 wells at 30 min after chlorination (33 mg tablet in 10 L water). (DOCX)

S4 Table Baseline characteristics in households that completed study vs. were lost to follow-up. (DOCX)

S5 Table Baseline characteristics by study group among households that completed the study. (DOCX)

S6 Table *E*. *coli* in tubewell and stored water by study group. (DOCX)

S7 Table Prevalence of diarrhea across study arms (2-day and 7-day recall period) among children 8–32 mo of age.^a^
(DOCX)

S8 Table Prevalence of negative control outcomes across study arms (2-day and 7-day recall period) among children 8–32 mo of age.^a^
(DOCX)

S1 Protocol Trial Protocol. (DOC)
