# Supplementary figures and images for: Effects of Source- versus Household Contamination of Tubewell Water on Child Diarrhea in Rural Bangladesh: A Randomized Controlled Trial
Source: PLoS One. 2015 Mar 27;10(3):e0121907. doi: 10.1371/journal.pone.0121907 (PMC4376788; doi:10.1371/journal.pone.0121907)

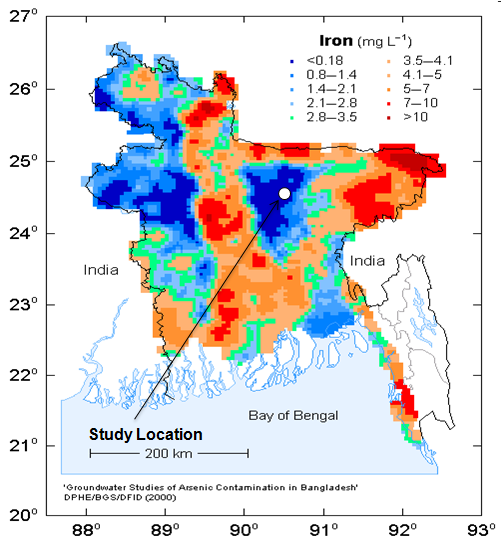

Supplement: S1 Fig — (TIF) [file pone.0121907.s002.tif]

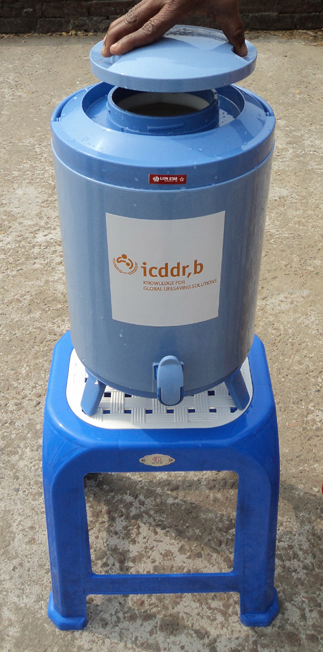

Supplement: S2 Fig — (TIF) [file pone.0121907.s003.tif]

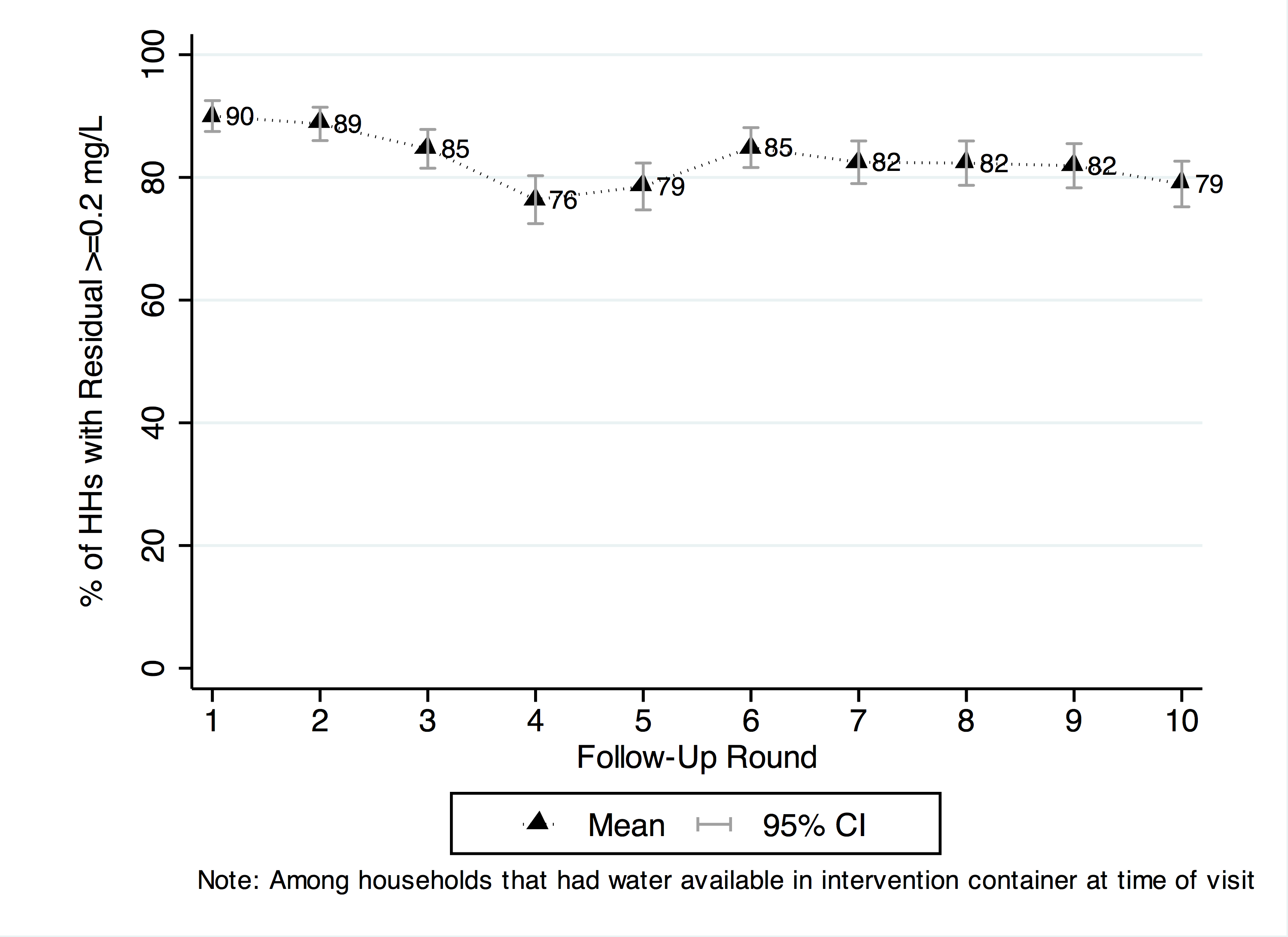

Supplement: S3 Fig — (TIF) [file pone.0121907.s004.tif]

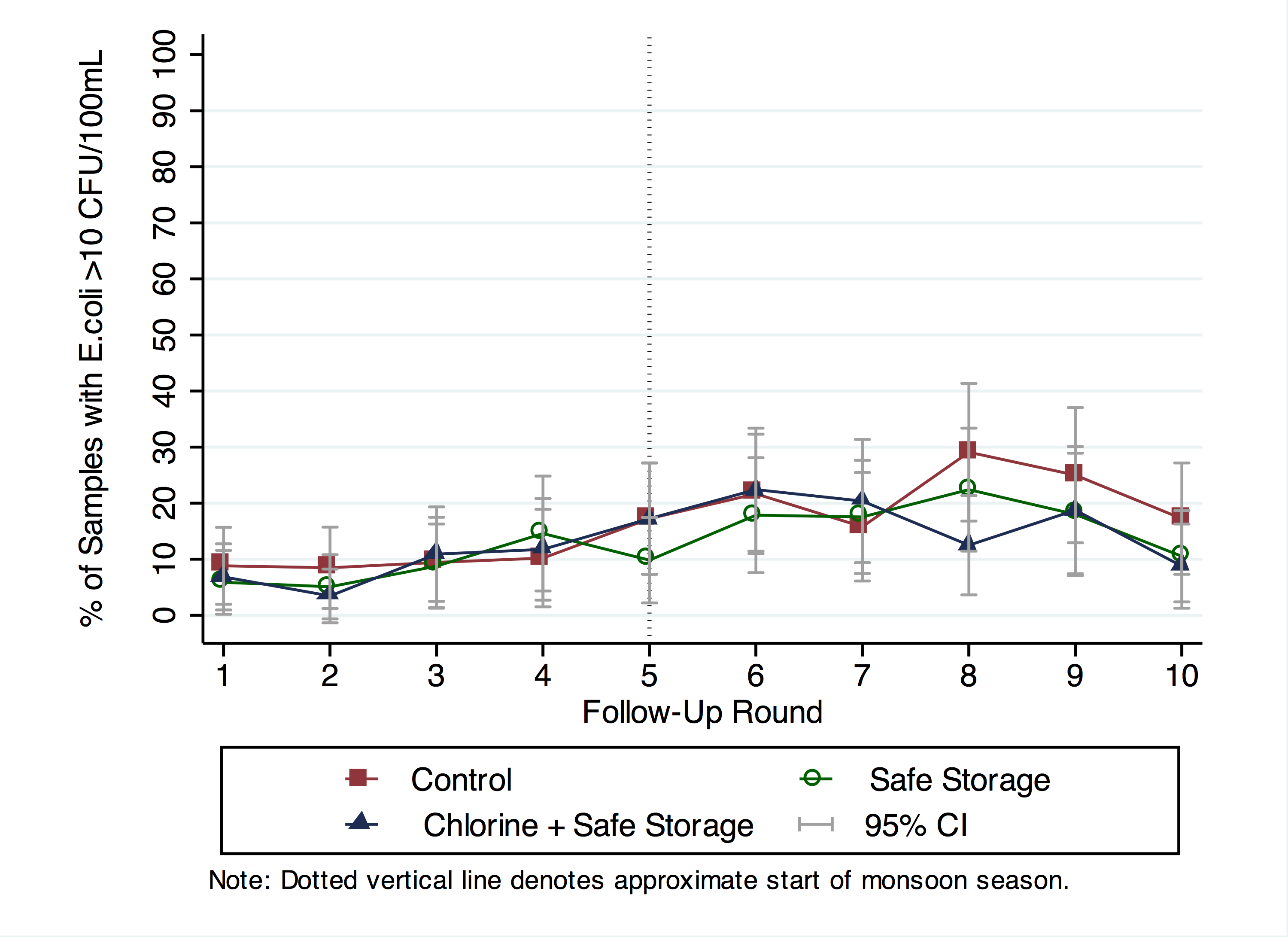

Supplement: S4 Fig — (TIF) [file pone.0121907.s005.tif]

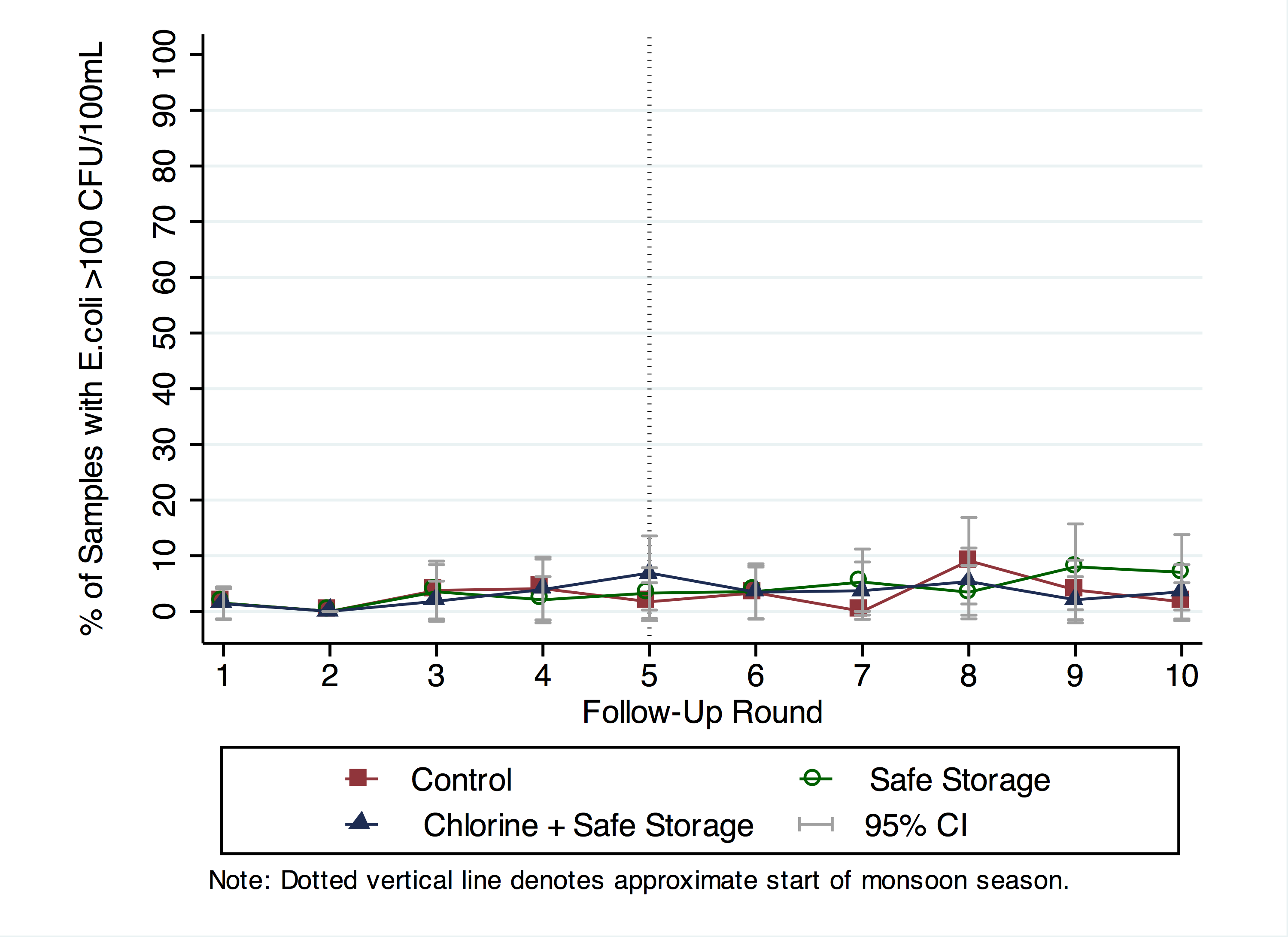

Supplement: S5 Fig — (TIF) [file pone.0121907.s006.tif]
