# Supplementary material for: Effects of Source- versus Household Contamination of Tubewell Water on Child Diarrhea in Rural Bangladesh: A Randomized Controlled Trial
Source: PLoS One. 2015 Mar 27;10(3):e0121907. doi: 10.1371/journal.pone.0121907 (PMC4376788; doi:10.1371/journal.pone.0121907)
Supplement: S1 Table — (DOCX) [file pone.0121907.s007.docx]

**S1 Table. Self-reported iron versus free chlorine residual among 52 wells at 30 min after chlorination**

| **Self-reported iron** | **Free chlorine residual <0.2 mg/L** | **Free chlorine residual ≥0.2 mg/L** | **Total** |
| --- | --- | --- | --- |
| Iron reported | 3 | 4 | 7 |
| Iron not reported | 0 | 45 | 45 |
| Total | 3 | 49 | 52 |
