# Supplementary material for: Effects of Source- versus Household Contamination of Tubewell Water on Child Diarrhea in Rural Bangladesh: A Randomized Controlled Trial
Source: PLoS One. 2015 Mar 27;10(3):e0121907. doi: 10.1371/journal.pone.0121907 (PMC4376788; doi:10.1371/journal.pone.0121907)
Supplement: S2 Table — (DOCX) [file pone.0121907.s008.docx]

**S2 Table. Sensitivity, specificity, positive predictive value (PPV) and negative predictive value (NPV) of self-reported iron complaints as predictor of free chlorine residual <0.2 mg/L vs. ≥0.2 mg/L among 52 wells at 30 min after chlorination**

| **Parameter** | **Value** |
| --- | --- |
| Sensitivity | 3/3 (100%) |
| Specificity | 45/49 (92%) |
| PPV | 3/7 (43%) |
| NPV | 45/45 (100%) |
