# Supplementary material for: Effects of Source- versus Household Contamination of Tubewell Water on Child Diarrhea in Rural Bangladesh: A Randomized Controlled Trial
Source: PLoS One. 2015 Mar 27;10(3):e0121907. doi: 10.1371/journal.pone.0121907 (PMC4376788; doi:10.1371/journal.pone.0121907)
Supplement: S3 Table — (DOCX) [file pone.0121907.s009.docx]

**S3 Table. Free chlorine residual among 52 wells at 30 min after chlorination (33 mg tablet in 10 L water)**

| **Free chlorine residual** | **Number (%) of wells** |
| --- | --- |
| <0.2 mg/L | 3 (6%) |
| 0.2 – 1 mg/L | 3 (6%) |
| 1 – 2 mg/L | 41 (79%) |
| >2 mg/L | 5 (9%) |
| Total | 52 (100%) |
