# Supplementary material for: Effects of Source- versus Household Contamination of Tubewell Water on Child Diarrhea in Rural Bangladesh: A Randomized Controlled Trial
Source: PLoS One. 2015 Mar 27;10(3):e0121907. doi: 10.1371/journal.pone.0121907 (PMC4376788; doi:10.1371/journal.pone.0121907)
Supplement: S4 Table — (DOCX) [file pone.0121907.s010.docx]

**S4 Table. Baseline characteristics in households that completed study vs. were lost to follow-up**

|  | **HHs that** | | **HHs lost to** | |
| --- | --- | --- | --- | --- |
|  | **completed study** | | **follow-up** | |
|  | **(N=1649 HHs)** | | **(N=151 HHs)** | |
|  | N | Mean/% | N | Mean/% |
| **Demographics and socioeconomics** |  |  |  |  |
| Number of index children 6-18 mo at enrollment | 1661 |  | 153 |  |
| Number of siblings 19-60 mo at enrollment | 363 |  | 33 |  |
| Mean age of respondent (years) | 1609 | 26 | 149 | 23 |
| Mean number of persons per HH | 1608 | 5.3 | 149 | 5.2 |
| Mean monthly HH income (USD) | 1589 | 93 | 149 | 96 |
| Mean number of rooms in HH | 1609 | 1.6 | 149 | 1.7 |
| Mean land owned by HH (acres) | 1599 | 0.5 | 145 | 0.6 |
| % of HHs with: |  |  |  |  |
| *Kaccha* walls ^a^ | 1609 | 35 | 149 | 32 |
| Electricity | 1608 | 36 | 149 | 31 |
| Cell phone | 1608 | 67 | 149 | 74 |
| TV | 1608 | 21 | 149 | 20 |
| % of mothers with 0 yrs of education | 1609 | 28 | 149 | 21 |
| **Water, sanitation and hygiene practices** |  |  |  |  |
| % of HHs with drinking water obtained: |  |  |  |  |
| Directly from tubewell | 1606 | 42 | 149 | 36 |
| From narrow-mouth container ^b^ | 1606 | 44 | 149 | 43 |
| From wide-mouth container | 1606 | 14 | 149 | 21 |
| % of HHs that treat drinking water | 1609 | 2 | 149 | 1 |
| % of HHs with: |  |  |  |  |
| Improved sanitation facility ^c^ | 1609 | 34 | 149 | 31 |
| Unimproved sanitation facility ^d^ | 1609 | 49 | 149 | 46 |
| No sanitation facility | 1609 | 17 | 149 | 23 |
| % of HHs where children <2 yrs defecate: |  |  |  |  |
| In latrine, potty or cloth | 1609 | 25 | 149 | 36 |
| In courtyard or living area | 1609 | 95 | 149 | 95 |
| Outside compound area | 1609 | 6 | 149 | 3 |
| % of HHs with: |  |  |  |  |
| Handwashing station (HWS) | 1608 | 81 | 149 | 81 |
| HWS <10 steps from latrine | 1608 | 33 | 149 | 32 |
| HWS with water | 1608 | 72 | 149 | 68 |
| HWS with soap | 1608 | 34 | 149 | 40 |
| **Health indicators in index children (6-18 mo at enrollment)** | | |  |  |
| Two-day % prevalence of: |  |  |  |  |
| Diarrhea | 1661 | 10 | 153 | 11 |
| Skin rash | 1661 | 15 | 152 | 15 |
| Ear infection | 1659 | 5 | 152 | 9 |
| Seven-day % prevalence of: |  |  |  |  |
| Diarrhea | 1661 | 15 | 153 | 16 |
| Skin rash | 1661 | 16 | 152 | 19 |
| Ear infection | 1659 | 5 | 152 | 9 |

HH: Household; USD: US dollars; HWS: Handwashing station

^a^ *Kaccha* walls refer to natural wall materials including jute, bamboo and mud. ^b^ The narrow-mouth containers used by all 3 groups were almost exclusively *kolshis*, which have a narrow mouth but a wide brim and no lid, allowing contamination. ^c^ Improved facilities include flush/pour flush latrines that drain to piped sewer, septic tank, or off-set pit; pit latrines with slab and water seal or with slab, no water seal but lid; and composting toilets. ^d^ Unimproved facilities include flush/pour flush latrines that drain into the environment; open pits; pit latrines without slab; pit latrines with slab but no water seal and no lid; and hanging toilets.
