# Supplementary material for: Effects of Source- versus Household Contamination of Tubewell Water on Child Diarrhea in Rural Bangladesh: A Randomized Controlled Trial
Source: PLoS One. 2015 Mar 27;10(3):e0121907. doi: 10.1371/journal.pone.0121907 (PMC4376788; doi:10.1371/journal.pone.0121907)
Supplement: S5 Table — (DOCX) [file pone.0121907.s011.docx]

**S5 Table. Baseline characteristics by study group among households that completed the study**

|  |  |  |  | |  | **Chlorine +** | |
| --- | --- | --- | --- | --- | --- | --- | --- |
|  | **Control** | | **Safe storage** | | | **safe storage** | |
|  | **(N = 565 HHs)** | | **(N = 547 HHs)** | | | **(N = 537 HHs)** | |
|  | N | Mean/% | N | Mean/% | | N | Mean/% |
| **Demographics and socioeconomics** |  |  |  | |  |  |  |
| Number of index children 6-18 mo at enrollment | 568 |  | 550 | |  | 543 |  |
| Number of siblings 19-60 mo at enrollment | 124 |  | 119 | |  | 120 |  |
| Mean age of respondent (years) | 550 | 26 | 534 | | 26 | 525 | 26 |
| Mean number of persons per HH | 550 | 5.3 | 534 | | 5.4 | 524 | 5.4 |
| Mean monthly HH income (USD) | 539 | 92 | 530 | | 94 | 520 | 93 |
| Mean number of rooms in HH | 550 | 1.7 | 534 | | 1.6 | 525 | 1.6 |
| Mean land owned by HH (acres) | 544 | 0.5 | 531 | | 0.4 | 524 | 0.5 |
| % of HHs with: |  |  |  | |  |  |  |
| *Kaccha* walls ^a^ | 550 | 34 | 534 | | 36 | 525 | 36 |
| Electricity | 550 | 33 | 534 | | 37 | 524 | 36 |
| Cell phone | 550 | 68 | 534 | | 67 | 524 | 66 |
| TV | 550 | 21 | 534 | | 23 | 524 | 19 |
| % of mothers with 0 yrs of education | 550 | 29 | 534 | | 28 | 525 | 28 |
| **Water, sanitation and hygiene practices** |  |  |  | |  |  |  |
| % of HHs with drinking water obtained: |  |  |  | |  |  |  |
| Directly from tubewell | 548 | 42 | 534 | | 41 | 524 | 43 |
| From narrow-mouth container ^b^ | 548 | 45 | 534 | | 44 | 524 | 42 |
| From wide-mouth container | 548 | 12 | 534 | | 14 | 524 | 15 |
| % of HHs that treat drinking water | 550 | 2 | 534 | | 2 | 525 | 1 |
| % of HHs with: |  |  |  | |  |  |  |
| Improved sanitation facility ^c^ | 550 | 32 | 534 | | 38 | 525 | 34 |
| Unimproved sanitation facility ^d^ | 550 | 52 | 534 | | 47 | 525 | 48 |
| No sanitation facility | 550 | 16 | 534 | | 16 | 525 | 19 |
| % of HHs where children <2 yrs defecate: |  |  |  | |  |  |  |
| In latrine, potty or cloth | 550 | 23 | 534 | | 25 | 525 | 26 |
| In courtyard or living area | 550 | 96 | 534 | | 94 | 525 | 94 |
| Outside compound area | 550 | 5 | 534 | | 7 | 525 | 6 |
| % of HHs with: |  |  |  | |  |  |  |
| Handwashing station (HWS) | 550 | 80 | 534 | | 81 | 524 | 81 |
| HWS <10 steps from latrine | 550 | 31 | 534 | | 34 | 524 | 33 |
| HWS with water | 550 | 72 | 534 | | 73 | 524 | 72 |
| HWS with soap | 550 | 32 | 534 | | 37 | 524 | 33 |
| **Health indicators in index children (6-18 mo at enrollment)** | | |  | |  |  |  |
| Two-day % prevalence of: |  |  |  | |  |  |  |
| Diarrhea | 568 | 10 | 550 | | 11 | 543 | 10 |
| Skin rash | 568 | 14 | 550 | | 15 | 543 | 15 |
| Ear infection | 568 | 4 | 549 | | 5 | 542 | 5 |
| Seven-day % prevalence of: |  |  |  | |  |  |  |
| Diarrhea | 568 | 15 | 550 | | 16 | 543 | 15 |
| Skin rash | 568 | 15 | 550 | | 16 | 543 | 16 |
| Ear infection | 568 | 5 | 549 | | 6 | 542 | 6 |

HH: Household; USD: US dollars; HWS: Handwashing station

^a^ *Kaccha* walls refer to natural wall materials including jute, bamboo and mud. ^b^ The narrow-mouth containers used by all 3 groups were almost exclusively *kolshis*, which have a narrow mouth but a wide brim and no lid, allowing contamination. ^c^ Improved facilities include flush/pour flush latrines that drain to piped sewer, septic tank, or off-set pit; pit latrines with slab and water seal or with slab, no water seal but lid; and composting toilets. ^d^ Unimproved facilities include flush/pour flush latrines that drain into the environment; open pits; pit latrines without slab; pit latrines with slab but no water seal and no lid; and hanging toilets.
