# Supplementary material for: Effects of Source- versus Household Contamination of Tubewell Water on Child Diarrhea in Rural Bangladesh: A Randomized Controlled Trial
Source: PLoS One. 2015 Mar 27;10(3):e0121907. doi: 10.1371/journal.pone.0121907 (PMC4376788; doi:10.1371/journal.pone.0121907)
Supplement: S6 Table — (DOCX) [file pone.0121907.s012.docx]

**S6 Table. *E. coli* in tubewell and stored water by study group**

|  |  | |  | | | **Chlorine +** | | |  | |  |  |  |  |
| --- | --- | --- | --- | --- | --- | --- | --- | --- | --- | --- | --- | --- | --- | --- |
|  | **Control** | | **Safe storage** | | | **safe storage** | | | **All arms** | |  |  |  |  |
|  | N | % | N | % |  | N | % |  | N | % |  |  |  |  |
| **Tubewell water (% of samples exceeding threshold)** | | | | | | | | |  |  |  |  |  |  |
| Year-round |  |  |  |  |  |  |  |  |  |  |  |  |  |  |
| > 0 CFU/100 mL | 576 | 44 | 576 | 38 | * | 574 | 40 |  | 1726 | 41 |  |  |  |  |
| > 10 CFU/100mL ^a^ | 569 | 16 | 571 | 13 |  | 568 | 13 |  | 1708 | 14 |  |  |  |  |
| > 100 CFU/100mL ^a^ | 569 | 3 | 571 | 4 |  | 568 | 3 |  | 1708 | 3 |  |  |  |  |
| Dry season |  |  |  |  |  |  |  |  |  |  |  |  |  |  |
| > 0 CFU/100 mL | 289 | 37 | 293 | 28 | * | 295 | 35 |  | 877 | 33 |  |  |  |  |
| > 10 CFU/100mL ^a^ | 287 | 11 | 293 | 9 |  | 295 | 10 |  | 875 | 10 |  |  |  |  |
| > 100 CFU/100mL ^a^ | 287 | 2 | 293 | 2 |  | 295 | 3 |  | 875 | 2 |  |  |  |  |
| Wet season |  |  |  |  |  |  |  |  |  |  |  |  |  |  |
| > 0 CFU/100 mL | 287 | 52 | 283 | 49 |  | 279 | 46 |  | 849 | 49 |  |  |  |  |
| > 10 CFU/100mL ^a^ | 282 | 22 | 278 | 17 |  | 273 | 16 |  | 833 | 18 |  |  |  |  |
| > 100 CFU/100mL ^a^ | 282 | 4 | 278 | 5 |  | 273 | 4 |  | 833 | 4 |  |  |  |  |
| **Stored water (% of samples exceeding threshold)** | | | | | | | | |  |  |  |  |  |  |
| Year-round |  |  |  |  |  |  |  |  |  |  |  |  |  |  |
| > 0 CFU/100 mL | 531 | 89 | 585 | 70 | * | 560 | 26 | ** | 1676 | -- |  |  |  |  |
| > 10 CFU/100mL ^a^ | 520 | 61 | 583 | 27 | * | 558 | 9 | ** | 1661 | -- |  |  |  |  |
| > 100 CFU/100mL ^a^ | 520 | 21 | 583 | 7 | * | 558 | 2 | ** | 1661 | -- |  |  |  |  |
| Dry season |  |  |  |  |  |  |  |  |  |  |  |  |  |  |
| > 0 CFU/100 mL | 270 | 84 | 303 | 59 | * | 292 | 17 | ** | 865 | -- |  |  |  |  |
| > 10 CFU/100mL ^a^ | 266 | 47 | 303 | 20 | * | 292 | 5 | ** | 861 | -- |  |  |  |  |
| > 100 CFU/100mL ^a, b^ | 266 | 13 | 303 | 5 | * | 292 | 1 | ** | 861 | -- |  |  |  |  |
| Wet season |  |  |  |  |  |  |  |  |  |  |  |  |  |  |
| > 0 CFU/100 mL | 261 | 94 | 282 | 82 | * | 268 | 35 | ** | 811 | -- |  |  |  |  |
| > 10 CFU/100mL ^a^ | 254 | 75 | 280 | 33 | * | 266 | 13 | ** | 800 | -- |  |  |  |  |
| > 100 CFU/100mL ^a^ | 254 | 29 | 280 | 9 | * | 266 | 3 | ** | 800 | -- |  |  |  |  |

CFU: Colony forming units

^a^ N different from N for >0 CFU/100 mL because of confluent (positive uncountable) plates. ^b^ Fisher’s exact test used to compare the chlorine + safe storage arm to other two arms due to sparse cells (<5 obs/cell).

* p-value <0.05 in comparison against control group using χ^2^ or Fisher’s exact test.

** p-value <0.05 in comparison against control group and safe storage group using χ^2^ or Fisher’s exact test.
