# Supplementary material for: Effects of Source- versus Household Contamination of Tubewell Water on Child Diarrhea in Rural Bangladesh: A Randomized Controlled Trial
Source: PLoS One. 2015 Mar 27;10(3):e0121907. doi: 10.1371/journal.pone.0121907 (PMC4376788; doi:10.1371/journal.pone.0121907)
Supplement: S7 Table — (DOCX) [file pone.0121907.s013.docx]

**S7 Table. Prevalence of diarrhea across study arms (2-day and 7-day recall period) among children 8-32 mo of age ^a^**

|  | **Control** | | **Safe storage** | | | | | **Chlorine + safe storage** | | | | | | | |
| --- | --- | --- | --- | --- | --- | --- | --- | --- | --- | --- | --- | --- | --- | --- | --- |
|  | N | Prev % | N | Prev % | PR^b^ | 95% CI | | N | Prev % | PR^b^ | 95% CI | | PR^c^ | 95% CI | |
| 2-day recall | 5654 | 6.8 | 5592 | 4.4 | 0.65 | (0.54, | 0.77) | 5505 | 4.4 | 0.65 | (0.54, | 0.77) | 1.00 | (0.83, | 1.21) |
| 7-day recall | 5654 | 10.6 | 5592 | 7.3 | 0.69 | (0.60, | 0.80) | 5505 | 6.7 | 0.64 | (0.55, | 0.73) | 0.92 | (0.79, | 1.08) |

^a^ Index children 8-32 mo of age during follow-up (6-18 mo at enrollment). ^b^ Prevalence ratio refers to comparison against control group. ^c^ Prevalence ratio refers to comparison against safe storage group.
