# Supplementary material for: Effects of Source- versus Household Contamination of Tubewell Water on Child Diarrhea in Rural Bangladesh: A Randomized Controlled Trial
Source: PLoS One. 2015 Mar 27;10(3):e0121907. doi: 10.1371/journal.pone.0121907 (PMC4376788; doi:10.1371/journal.pone.0121907)
Supplement: S8 Table — (DOCX) [file pone.0121907.s014.docx]

**S8 Table.** **Prevalence of negative control outcomes across study arms (2-day and 7-day recall period) among children 8-32 mo of age ^a^**

|  | **Control** | | **Safe storage** | | | | | **Chlorine + safe storage** | | | | | | | |
| --- | --- | --- | --- | --- | --- | --- | --- | --- | --- | --- | --- | --- | --- | --- | --- |
|  | N | Prev % | N | Prev % | PR ^a^ | 95% CI | | N | Prev % | PR^a^ | 95% CI | | PR^b^ | 95% CI | |
| 2-day recall |  |  |  |  |  |  |  |  |  |  |  |  |  |  |  |
| Skin rash | 5655 | 3.9 | 5592 | 3.5 | 0.89 | (0.67, | 1.17) | 5505 | 3.3 | 0.86 | (0.65, | 1.12) | 0.96 | (0.73, | 1.28) |
| Ear infection | 5655 | 1.7 | 5590 | 2.3 | 1.38 | (0.88, | 2.17) | 5504 | 1.7 | 1.03 | (0.64, | 1.64) | 0.74 | (0.47, | 1.17) |
| 7-day recall |  |  |  |  |  |  |  |  |  |  |  |  |  |  |  |
| Skin rash | 5655 | 4.4 | 5592 | 3.8 | 0.88 | (0.68, | 1.14) | 5505 | 3.7 | 0.86 | (0.66, | 1.10) | 0.97 | (0.75, | 1.26) |
| Ear infection | 5655 | 2.1 | 5590 | 2.6 | 1.26 | (0.84, | 1.91) | 5504 | 1.9 | 0.94 | (0.61, | 1.45) | 0.74 | (0.48, | 1.14) |

^a^ Index children 8-32 mo of age during follow-up (6-18 mo at enrollment). ^b^ Prevalence ratio refers to comparison against control group. ^c^ Prevalence ratio refers to comparison against safe storage group.
