# Supplementary material for: Effects of Source- versus Household Contamination of Tubewell Water on Child Diarrhea in Rural Bangladesh: A Randomized Controlled Trial
Source: PLoS One. 2015 Mar 27;10(3):e0121907. doi: 10.1371/journal.pone.0121907 (PMC4376788; doi:10.1371/journal.pone.0121907)
Supplement: S1 Protocol — (DOC) [file pone.0121907.s015.doc]

**S1 Protocol.**

**“Health impact of treating and safely storing shallow tubewell drinking water”**

## Hypothesis to be Tested:

Please briefly list the Hypothesis to be tested and provide the scientific basis of the hypothesis, critically examining the observations leading to the formulation of the hypothesis.

Drinking water from shallow tubewells that are intermittently contaminated with enteric pathogens contributes importantly to diarrhea among children in rural Bangladesh.

##

## Specific Aims:

Describe the specific aims of the proposed study. State the specific parameters, biological functions, rates, processes etc. that will be assessed by specific methods.

In a typical setting in rural Bangladesh where households use shallow tubewell water for drinking:

1. Evaluate the impact of chlorination and safe storage of drinking water on childhood diarrhea compared to standard water handling habits and practices during the dry as well as wet season.
2. Evaluate the impact of safe storage of drinking water on childhood diarrhea separate from chlorination of tubewell water during the dry as well as wet season.

##

## Background of the Project including Preliminary Observations

Provide relevant background of the proposed study, and discuss the previous works on the research topic by citing specific references. Describe in a logical way how the present hypothesis is supported by the relevant background observations including any preliminary results that may be available. Provide scientific validity of the hypothesis on the basis of background information Critically analyze available knowledge in the field of the proposed study and discuss the questions and gaps in the knowledge that need to be fulfilled to achieve the proposed goals.. If there is no sufficient information on the subject, indicate the need to develop new knowledge. Also include the significance and rationale of the proposed work by specifically discussing how these accomplishments will bring benefit to human health in relation to biomedical, social, and environmental perspectives.

The World Health Organization estimates that 1.8 million children die annually from diarrheal disease (World Health Organization, 2005). In settings where drinking water is highly contaminated with human fecal organisms, water treatment at point of use that consistently removes microbiological indicators of fecal contamination, consistently and substantially reduces childhood diarrhea (Clasen et al., 2007b, Fewtrell et al., 2005, Arnold and Colford, 2007). These large reductions in diarrheal incidence are noted even in settings where the sole intervention is improvement in the microbiological quality of drinking water at point of use, without any intervention to improve handwashing or sanitation (Fewtrell et al., 2005, Luby et al., 2006b).

There is much less data available on the relationship between low levels of microbiological contamination of drinking water and the risk of childhood diarrhea (Moe et al., 1991). The World Health Organization Guidelines for Drinking Water Quality recommends reducing exposure to pathogens in raw water to reduce the disease burden to 10^-6^ disability adjusted life year (DALY) per person per year (World Health Organization, 2004). However, the lack of data on the marginal health impact of further treatment of water with low levels of fecal indicator organisms, means that the relative value of further improvements in water quality are unclear, especially in settings where hygiene practices and sanitation are compromised, and would benefit from focused intervention.

When shallow tubewells replaced highly contaminated surface water as the primary source of drinking water in Bangladesh in the late 20th century, contemporary studies demonstrated no reduction in diarrheal disease with this improvement in water source (Levine et al., 1976, Curlin et al., 1977, Sommer and Woodward, 1972, Khan et al., 1978, Briscoe, 1978). Some observers have argued that this lack of reduction in diarrhea following introduction of tubewells is likely a result of continued exposure to enteric pathogens from other sources (Levine et al., 1976, Briscoe, 1978). The lack of health improvements in transitioning to tubewells is consistent with the lack of significant health gains observed in intervention studies focusing on water quality improvements at the source (Fewtrell et al., 2005). Thus, one hypothesis to explain the failure of shallow tube wells to prevent diarrhea in Bangladesh, is that the resulting tubewell water remains too contaminated with enteric pathogens. Recent studies, including studies conducted by this research group, confirm that up to 40% of tubewells at any one time in various regions of Bangladesh are contaminated with thermotolerant coliforms or *Escherichia coli* (Hoque, 1999, Islam et al., 2001, Luby et al., 2006a, Hoque et al., 2006). However, the level of contamination is generally low. Among 207 tubewell water samples collected from 3 flood prone districts in Bangladesh in 2005, 29% were contaminated with thermotolerant coliforms (median 5 colony forming units/100ml, n=60), and 13% with *E. coli* (median 5 colony forming units/100ml, n=27) (Luby et al., 2008). The World Health Organization guidelines define safe drinking water as water that has less than 1 colony forming unit of *E. coli* per 100ml, and it is unclear if the level of contamination typically observed in tubewells in Bangladesh has a substantial impact on health. An alternative hypothesis to explain the failure of tubewells in reducing diarrhea is that even when relatively clean source water is available, contamination during collection, storage and handling may again put children at risk of diarrhea (Trevett et al., 2004),

In contrast to interventions to improve water quality at the source, high quality intervention studies that improve water quality at the point of use through treatment of water in households show a 39% reduction in diarrhea (Fewtrell et al., 2005). Point of use water treatment typically produces water that is microbiologically less contaminated than water source improvements (Mintz et al., 2001). Moreover, storing water safely in the home by using storage containers with lids and spigots has been shown to result in improvements in health (Mintz et al., 1995, Roberts et al., 2001). The magnitude of the risk of diarrhea due to consumption of tubewell water in the rural Bangladesh setting is not defined, and so the relative importance of further water quality improvements through treatment and safe storage in households is unknown. An estimated 16,600 – 27,700 children die each year in Bangladesh from diarrheal disease (Tanaka et al., 2007). Since more than 90% of households in rural Bangladesh use tubewells as their source of drinking water (NIPORT, 2005), the question of the marginal benefit of further treatment for tubewell water is an important issue for child health in Bangladesh.

In the context of rural Bangladesh, seasons often affect tubewell water quality through rainfall patterns, with 9% of very shallow tubewells (<20 m) shown to be contaminated with E.coli during the dry season from November through April and 61% contaminated during the rainy season from May until the end of October (Leber et al. 2010). Seasonality also has an impact on water retrieval, storage and handling practices. Some shallow tubewells run dry during the winter months and families shift to alternative sources, such as irrigation pumps that draw water from deeper aquifers or deep tubewells that are located further away from the compound. Recent data from rural Fulbaria suggest that, over the course of the dry season, roughly 30% of respondents have stopped using the shallow tubewell that was their primary drinking water source at the onset of the dry season. It is therefore expected that the magnitude of the health risk from microbiological contamination of drinking water and consequently the effectiveness of water treatment and safe storage interventions will show seasonal variation.

Tubewell water in Bangladesh is also frequently contaminated with arsenic. In a survey in 41 of the 64 districts in Bangladesh, 35% of tested tubewells were contaminated with arsenic over the Bangladesh standard of 50 μg/L and 51% were contaminated with arsenic over the World Health Organization (WHO) standard of 10 μg/L (BGS and DPHE, 2001). Strategies to reduce the population’s exposure to arsenic include filters and other strategies for removing arsenic from tubewell water, however, such approaches often worsen microbial contamination (Norton et al., 2009, Howard et al., 2006). With multiple threats to the water supply in Bangladesh including increased population pressures, global warming, and scheduled dam construction by India and China on major rivers that flow into Bangladesh, understanding how best to provide safe drinking water is an issue of growing importance for child health in Bangladesh.

This question also has global implications (Luby, 2007). Currently, the global standard for safe water is an assessment of the proportion of the population that has access to an “improved water supply.”(UN Millenium Project Task Force on Water and Sanitation, 2005, United Nations, 2006). However, “improved” water supplies are often contaminated with human fecal bacteria (Luksamijarulkul et al., 1994, Jiwa et al., 1991, Nogueira et al., 2003, Vollaard et al., 2005), and so the health benefit of reaching the Millennium Development Targets for safe water may be limited.

## Research Design and Methods

Describe in detail the methods and procedures to be used in accomplishing the objectives and specific aims of the project. Discuss the alternative methods that are available and justify the use of the method proposed in the study. Justify the scientific validity of the methodological approach (biomedical, social, or environmental) as an investigation tool to achieve the specific aims. Discuss the limitations and difficulties of the proposed procedures and sufficiently justify the use of them. Discuss the ethical issues related to biomedical and social research for employing special procedures, such as invasive procedures in sick children, use of isotopes or any other hazardous materials, or social questionnaires relating to individual privacy. Point out safety procedures to be observed for protection of individuals during any situations or materials that may be injurious to human health. The methodology section should be sufficiently descriptive to allow the reviewers to make valid and unambiguous assessment of the project.

**Setting:** We will conduct the study in the Fulbaria upazila of Mymensingh district, where groundwater chemistry has been proven to be amenable to disinfection with chlorine. Groundwater in many areas in Bangladesh is rich in iron and other ions that react with chlorine and limit its capacity to provide adequate disinfection. Groundwater testing in Fulbaria has shown that chlorination works effectively at this location, as discussed below.

**Develop Intervention:** This is an efficacy study, i.e. it is trying to answer the question: if tubewell water were effectively chlorinated and/or safely stored in homes, what would be the impact on childhood diarrhea. To efficiently answer this question, we will strive to optimize the use of the intervention in those households randomized to receive the intervention. Chlorinating water has been shown to be an effective way of disinfecting water in the home (Arnold and Colford, 2007). We will use Aquatabs for chlorination, which are effervescent water purification tablets that utilize sodium dichloroisocyanurate (NaDCC) as the chlorine donor. NaDCC was judged to be a safe and appropriate treatment for water by the World Health Organization (Clasen and Edmondson, 2006) and has been tested and found acceptable to a community of urban residents in Dhaka, Bangladesh (Clasen et al., 2007a). Aquatabs are also easier to store, handle and correctly dose than liquid forms of chlorine (Clasen and Edmondson, 2006).

**Groundwater Testing:** To optimize the use of Aquatabs and determine the appropriate dose to be assigned to study participants, we collected water samples from 52 shallow tubewells geographically spread out over all 13 unions in Fulbaria. Water was collected in 5L and 10L jerry cans with a narrow mouth and lid. One Aquatabs tablet was added to each vessel, resulting in a free chlorine dose of 2 mg/L in the 10L container and 4 mg/L in the 5L container. Remaining chlorine residual in the vessels was measured half an hour and 24 hours after the addition of the tablet to assess whether it meets the target criteria of having a minimum residual of 0.2 mg/L at 24 hours (to ensure adequate disinfection) and a maximum residual of 2 mg/L at half an hour (to minimize taste and odor concerns). It should be noted that the 2 mg/L taste/odor threshold we are targeting is well under the WHO health limit of 5 mg/L. Based on the testing results, we determined that using one Aquatabs with a 10L jerry can provides the optimal chlorine dose in Fulbaria.

**Pilot Study:** Having determined the optimal chlorine dose, we will conduct a pilot study to develop the optimal promotion strategy to ensure high uptake of the tablets by study participants. The pilot study will entail 80 households with children between the ages of six months and two years. All households will receive a 10L safe water storage container and half of the households will also receive chlorine tablets. Moreover, half of the 80 households will be randomized to receive intensive promotion of the interventions via weekly household visits, and the other half will be randomized to receive less intensive promotion via monthly visits. After informed consent has been obtained (see Appendix 1B for consent form), the baseline questionnaire will be administered.at the first visit and the interventions will be introduced to the households. The intensive promotion group will be visited once a week for a month to reinforce the promotion, and the less intensive promotion group will be visited only at the end of the month. At each household visit, chlorine residual in the storage container will be measured and qualitative information will be collected about the participants’ satisfaction with the tablets. At the last visit, the follow-up questionnaire will also be administered.

The pilot will help us develop the optimum promotion strategy for introducing the interventions to the full study population and assess the uptake and acceptability of the products. However, previous piloting efforts by other teams suggest that ensuring good uptake with chlorine may be challenging due to the unfamiliar taste/smell of chlorine. We therefore propose to also consider a filter that has been proven to be microbiologically effective as a potential back-up and assess if the uptake of filters is significantly better than chlorine. We are currently in the process of procuring the filters and if they become available during the pilot period, we propose enrolling an additional 20 households into the pilot study and providing them with filters to assess if the users strongly prefer these over chlorine tablets. The rest of the protocol has been written assuming that we will use Aquatabs in the full-scale study, but depending on the results of the pilot, we may reconsider this decision if filters prove to have significantly better user uptake. If we do decide to use filters instead of Aquatabs in the full-scale study, we will amend the protocol accordingly.

The filter we are proposing to evaluate is manufactured by PATH, an international NGO that works in developing countries on water treatment as well as other public health issues. Previous piloting efforts with locally available filters by other teams suggested that these were vulnerable to re-contamination of the water after treatment as they have several detachable plastic parts that can become contaminated due to improper maintenance and cleaning practices. The filters manufactured by PATH have a compact design with a ceramic filter housed inside a robust plastic casing with a lid, minimizing the risk of contamination. They are easy to use with minimal instruction and maintenance requirements and have been specifically designed to be used in developing country settings.

**Intervention Groups:** The study will have the following three arms, which will allow us to establish the respective roles of household water treatment and safe water storage in preventing diarrheal illness in children in the rural Bangladesh setting.

Group 1 – Aquatabs and Safe Storage Vessel: Households in this group will receive Aquatabs for water purification, a safe water storage container to prevent contamination during storage in the home, and training and encouragement to treat and safely store their water using the provided products.

Group 2 – Safe Storage Vessel: Households in this group will receive a safe water storage container, and training and encouragement to safely store their water using the provided products. If our study shows that treatment of tubewell water at the household level is effective in protecting children’s health, they will receive a six-month supply of water treatment tablets at the end of the study.

Group 3 – Standard Practices: Households in this group will not receive any water treatment or storage intervention during the study. They will continue their usual water collection and storage practices. If our study shows that treatment and safe storage of tubewell water at the household level is effective in protecting children’s health, they will receive the same safe water storage container as Groups 1 and 2 as well as a six-month supply of water treatment tablets at the end of the study.

**Selection of Households:** Households to participate in the study will be recruited through a systematic sample from a subset of villages in Fulbaria selected based on field factors such as ease of transport and proximity to the field office. The inclusion criteria will include (1) using a shallow tubewell that is free from iron complaints as the bari’s primary source of drinking water and (2) having a child between the ages of six months and two years living in the bari. The primary caregiver in the household that has the child in the target range will be recruited into the study. If there are multiple households in the bari that have a child between the ages of six months and two years, one of the households will be randomly selected and the primary caregiver in the selected household will be recruited. Recruited households will be randomized into one of the three study arms by block randomization through a randomly generated, pre-established sequence that will determine which intervention will be allocated to the household.

**Promotion of Interventions:** The intervention will continue for ten months. At the first visit to the households, Groups 1 and 2 will be provided with a plastic water storage container with a lid and a narrow mouth/tap, and Group 1 will also be given a one-month supply of tablets. Households will be taught through demonstrations and a flipchart with illustrations how to use the tablets as well as how to properly use and clean the safe storage containers, and they will be encouraged to use them regularly. They will also be given an instructions sheet with illustrations that will be left at a visible spot in the household and serve as a reminder to clean the containers and if applicable add the water treatment tablets. A field research assistant who is not part of the evaluation team will visit the household once per month to encourage regular water treatment and safe storage, replenish the supply of tablets and address any issues with the maintenance and cleaning of the safe storage vessels. The rest of the protocol has been written assuming that households will receive monthly promotion visits. However, if the pilot study shows that more frequent, intensive promotion leads to significantly better uptake, in the full-scale study we will consider replacing the monthly promotion frequency with more frequent promotion visits.

**Data Collection:** There will be one baseline visit and ten follow-up visits to participating households.

Baseline Visit: We will conduct a baseline survey including demographics, household description, socioeconomic status, pre-intervention water, sanitation and hygiene habits and baseline levels of diarrhea in children between the ages of six months and two years. If there are any children in a recruited household that are between the ages of two and five, we will collect diarrhea data on them as well to allow comparison of our results to other findings in the literature that focus on children under the age of five. During the baseline visit, we will assess the microbial quality of the tubewell and stored water for each study household using the hydrogen sulphide (H2S) test. Tubewell and stored water samples from a subset of 150 households (50 from each study arm) will be analyzed for total coliforms and E. coli using membrane filtration.

Follow-Up Visits: Every month a field research assistant will visit each participating household to conduct a follow-up survey and collect information on the prevalence of diarrhea among children between the ages of six months and five years in the 48 hours as well as seven days preceding the visit. We are using a 48-hour recall because this typically provides a more valid estimate than longer recall periods which lead to under-reporting of health outcomes (Alam et al., 1989, Ramakrishnan et al., 1999, Feikin et al., 2010). We are also using a seven-day recall because recent findings indicate that while this longer recall period leads to only minimal bias in terms of the accuracy of recall it can result in significant gains of statistical power (Arnold et al., 2011).We will visit households once every month, because more frequent visits provide little additional statistical power due to correlation of repeated measures on the same individuals (Schmidt et al., 2007).

At each visit, we will also collect information on user compliance with the interventions by asking about type and timing of water treatment practices, counting the number of remaining tablets and inspecting the water storage facilities, as well as asking participants when and how they cleaned the storage container. We will also collect water samples from the tubewell and storage container in households to assess the microbial quality of the water, explore the extent of contamination at the source versus in the home and gauge the effectiveness of the interventions in preventing contamination. We will perform H2S testing in half of study households and total coliform/E.coli testing with membrane filtration for a subset of 150 households (50 from each study arm). Moreover, for each household in the Aquatabs group, we will analyze stored water samples for residual chlorine concentration to monitor compliance with the intervention.

**Laboratory Testing:** Water samples will be placed on ice and transported to the Fulbaria field office where the appropriate tests will be conducted by the field microbiologists. All testing will be performed promptly upon transporting the samples to the field office. The hydrogen sulfide (H2S) test is a simple low-cost test (Sobsey and Pfaender, 2003). Twenty ml of water will be added in H2S bottle and incubated at room temperature for 24-48 hours. If the colour of the water changes to black, it will indicate that the water is contaminated with coliform which will indicate that the water is unfit for human consumption. For membrane filtration, collected water samples will be quantitatively tested for total coliforms and E. coli using the DelAgua kit with MI Broth (Whatman Inc, New Jersey, USA). For residual chlorine testing, we will use the n,n-diethyl-p-phenylenediamine (DPD) colorimetric method which is part of the DelAgua kit.

**Study Timeline:**

## Sample Size Calculation and Outcome (Primary and Secondary) Variable(s)

Study Arms:

1: Chlorine + Safe Storage

2: Safe Storage

3: Standard Practice

Hypothesis 1 (Main Hypothesis, Arm 1 vs. 3): Children who live in households that use tubewell water for drinking that is treated with effective chlorination and safely stored will have less diarrhea than households that use shallow tubewell water for drinking that is not treated and not safely stored.

Hypothesis 2 (Arm 1 vs. 2): Children who live in households that use tubewell water for drinking that is treated with effective chlorination and safely stored will have less diarrhea than households that use tubewell water for drinking that is safely stored but not treated with chlorine.

Hypothesis 3 (Arm 2 vs. 3): Children who live in households that use tubewell water for drinking that is safely stored but not treated will have less diarrhea than households that use tubewell water for drinking that is not treated and not safely stored.

While hypothesis 1 (the combined effect of chlorination and safe storage) is our main hypothesis, the study has been conservatively sized to have sufficient power to test hypothesis 2 (individual effect of chlorination), using the following assumptions:

- 2-day prevalence (as opposed to 7-day prevalence) used in calculations to be conservative
  - 14% longitudinal prevalence in Arm 3 (based on SHEWA-B data)
  - 11.6% longitudinal prevalence in Arm 2 (based on Roberts et al., 2001)
  - 9.1% longitudinal prevalence in Arm 1 (based on Arnold and Colford, 2007)
- 1 child between the ages of six months and two years per tubewell
- 5 follow-up visits per child
- 0.13 ICC and 1.50 design effect for repeated measures per child
- 10% drop-out
- One-tailed test with 95% confidence level

There is firm evidence from previous studies on the protective effect of household water treatment with chlorine, justifying a one-tailed test. Based on these assumptions, a sample size of 575 households per arm will have 80% power to detect the difference between Arms 1 and 2. Because of the many uncertainties in the sample size estimation, we will enroll 600 households in the Aquatabs and safe storage group, 600 households in the safe storage only group and 600 households in the standard habits and practices control group. This conservative sample size allows us to detect the difference between Arms 1 and 3 and test our main hypothesis with 99% power with either a one-tailed or a two-tailed test.

## Facilities Available

Describe the availability of physical facilities at site of conduction of the study. For clinical and laboratory-based studies, indicate the provision of hospital and other types of adequate patient care and laboratory support services. Identify the laboratory facilities and major equipment that will be required for the study. For field studies, describe the field area including its size, population, and means of communications.

ICDDR.B has maintained a field office in Fulbaria that will serve as the base station for field activities. Laboratory analyses will be carried out in the field office through the use of field kits designed for carrying out such tests in the absence of a fully-equipped laboratory.

The Fulbaria upazila has an area of 99,436 acres and a population of 396,000. The upazila consists of 13 unions, 104 mouzas (administrative units) and 135 villages. Fulbaria sadar union is about 22 kilometers from Mymensingh sadar and about 120 kilometers from Dhaka ICDDR,B center by road.

## Data Safety Monitoring Plan (DSMP)

All clinical investigations (biomedical and behavioural intervention research protocols) should include the Data and Safety Monitoring Plan (DSMP) to provide the overall framework for the research protocol’s data and safety monitoring. It is not necessary that the DSMP covers all possible aspects of each element. When designing an appropriate DSMP, the following should be kept in mind.

1. All investigations require monitoring;
2. The benefits of the investigation should outweigh the risks;
3. The monitoring plan should commensurate with risk; and
4. Monitoring should be with the size and complexity of the investigation.

Safety monitoring is defined as any process during clinical trials that involves the review of accumulated outcome data for groups of patients to determine if any treatment procedure practiced should be altered or not.

The interventions planned for this study, chlorine treatment of drinking water, and storage of water in a container that prevents re-contamination have been widely used and found safe. The dosage of chlorine will be controlled by the use of a single tablet for a known volume of water. Even if households made an error in dosing, the doubled or tripled the chlorine concentration would still not represent a substantial risk to human health. In addition, at such a high dosage effect on taste and smell of the water would make it unpalatable.

Although we do not expect any adverse outcomes, we will assess the well-being of household participants at each monthly visit. Field workers will refer members of households with serious health problems to appropriate care providers. They will record any serious health problems and we will track these regularly to see if there is any association with any of the interventions. In addition, participating subjects will be able to contact the local research investigator if they have any concerns.

## Data Analysis

Describe plans for data analysis. Indicate whether data will be analysed by the investigators themselves or by other professionals. Specify what statistical software packages will be used and if the study is blinded, when the code will be opened. For clinical trials, indicate if interim data analysis will be required to determine further course of the study.

The primary outcome will be longitudinal prevalence of diarrhea among children between the ages of six months and two years. If there are any children in a recruited household that are between the ages of two and five, their diarrhea outcome will be considered as well. We will use longitudinal prevalence because it is more strongly associated with malnutrition and mortality than is diarrhea incidence (Morris et al., 1996). We will collect diarrhea over 1 day, 2 day and 7 day recall periods. We will use a 7 day recall period to increase statistical power (Schmidt et al. 2010) if we confirm that the ratio of diarrhea cases in the past 24 hours (1 day recall) to cases that terminated between days 2-7 (the CT ratio) does not differ by randomized treatment group (Boerma et al. 1991). If the CT ratio differs by treatment group, it will indicate differential recall bias and so we will use a more conservative 2 day recall period in our primary analysis. Using STATA and/or SAS for data analysis, we will make the following three comparisons:

- Group 1 vs. 2: We will compare the longitudinal prevalence of diarrhea among children between the ages of six months and two years in households that receive chlorine water treatment and safe storage to households that receive safe storage. This will assess the contribution of water disinfection (separate from safe storage) to any reduction in diarrhea.
- Group 2 vs. 3: We will compare the longitudinal prevalence of diarrhea among children between the ages of six months and two years in households that receive safe storage only to households that receive no intervention. This will assess the effect of safe storage.
- Group 1 vs. 3: We will compare the longitudinal prevalence of diarrhea among children between the ages of six months and two years in households that receive chlorine water treatment and safe storage to households that receive no intervention. This will assess the combined effects of safe storage and disinfection.

We will assess the magnitude of the differences in longitudinal prevalence by group by calculating a risk ratio between the groups. We will evaluate if risk ratios differ from 1.0 at traditional testing levels (*P*=0.05) using robust standard errors that account for repeated observations within children (Zeger and Liang 1986). We will repeat the calculations using the longitudinal prevalence of diarrhea among children under the age of five as the outcome measure to allow us to compare our results to other findings from the literature that focus on children under five.

Secondary outcomes will include the proportion of households by group that have contaminated water as measured by H2S testing and by quantitative evaluation of total coliforms and *E. coli.* We will assess the proportion of samples within each group that have no evidence of contamination. We will compare whether these proportions are different than would be expected by chance using chi square when there are no repeated samples and using general estimated equations when repeated samples are analyzed from the same household.

## Ethical Assurance for Protection of Human Rights

Describe the justifications for conducting this research in human participants. If the study needs observations on sick individuals, provide sufficient reasons for using them. Indicate how participants rights will be protected, and if there would be benefit or risk to each participants of the study.

This study enrols human subjects, because the study question addresses the issue of the impact of additional water treatment behavior on human health and the question can only be answered by enrolling human subjects. The objectives and study procedures will be explained to all candidate households and only households that provide informed consent will be enrolled. Households will be free to drop out of the study at any time.

Benefits to the study participants include that some participating families will receive water treatment and/or a water storage container. Moreover, if the study shows that treating tubewell water at the household level and storing it safely is effective in improving children’s health, the safe storage group and the non-intervention group will be given a six-month supply of chlorine tablets at the end of the study and the non-intervention group will also be given a safe storage container such that all study participants will ultimately have received water treatment tablets in addition to safe storage containers. At the end of providing free tablets, all three study groups will also be encouraged to continue to treat their water. If children are identified through the routine surveillance who appear seriously ill to do field research assistant, they will be referred to an appropriate healthcare facility.

If the participants consent to participate in the five additional follow-up and promotional visits, we will provide these intervention container and tablets to the control arm households after these additional visits. However, during reconsenting, we would provide all the participants color pencils/toys for their small kids. This would be a token of appreciation but not compensation, which will help for the intellectual development of their kids but has small monetary value.

The risks to study participants are few. Chlorine treatment of drinking water has been used for over a century and is widely recognized as a safe and appropriate way to treat drinking water. The specific chemical used for water treatment in this study, sodium dichloroisocyanurate, has been reviewed by the World Health Organization and judged to be safe (Clasen and Edmondson, 2006).

The control group will receive no water treatment during the study period, but will continue to be followed while they use their standard water collection habits and storage practices. This group risks being exposed to significant waterborne pathogens without treatment. If, over the course of the study, we encounter households in this group whose drinking water does not meet the WHO criteria for low risk (less than 10 CFU of thermotolerant coliforms per 100 mL), these households will be notified of their water quality results, informed about locally available water treatment methods and encouraged to use them. These include liquid bleach, bleaching powder and halotablets, as well as boiling. Moreover, this group is not being exposed to any increased risk by participating in the study. Indeed, their water collection and storage practices are the standard practices of tens of millions of Bangladesh households every day. We do not know if this low-level exposure to fecally contaminated drinking water leads to significant household diarrhea. Many commentators argue that low levels of contamination are not a significant health risk (Moe et al., 1991). The only way that we can learn whether increased efforts to improve the microbiological quality of drinking water will reduce diarrhea is by evaluating the intervention. This evaluation requires a non-intervention group for comparison.

## Use of Animals

Describe if and the type and species of animals to be used in the study. Justify with reasons the use of particular animal species in the experiment and the compliance of the animal ethical guidelines for conducting the proposed procedures.

Not applicable

## Minor changes made to this protocol before study implementation:

1. Random subset of households in randomly selected villages (instead of systematic subset of households in villages close to the field office) to achieve representative random sample.
2. 180 households (60 from each study arm) tested for *E. coli* and total coliforms instead of 150 households (50 from each study arm) to achieve testing in 10% of study households.
3. MI Agar (Becton, Dickinson and Company, Maryland, USA) to enumerate *E. coli* and total coliforms instead of MI Broth (Whatman Inc, New Jersey, USA).
4. Digital colorimeter (Hach, Colorado, USA) to measure free chlorine residual instead of DelAgua kit to achieve increased precision.
5. Longitudinal prevalence in the protocol used to refer to period prevalence. Period prevalence measured to avoid inaccuracies associated with collecting diarrhea data for each day of the 7-day recall period.

## Literature Cited

Identify all cited references to published literature in the text by number in parentheses. List all cited references sequentially as they appear in the text. For unpublished references, provide complete information in the text and do not include them in the list of Literature Cited. There is no page limit for this section, however, exercise judgment in assessing the “standard” length.
